# Cooperative ETS transcription factors are required for lymphatic endothelial cell integrity and resilience

**DOI:** 10.1172/JCI196119

**Published:** 2025-12-23

**Authors:** Myung Jin Yang, Seok Kang, Seon Pyo Hong, Hokyung Jin, Jin-Hui Yoon, Cheolhwa Jin, Chae Min Yuk, Lydia Getachew Gebeyehu, Junho Jung, Sung-Hwan Yoon, Hyuek Jong Lee, Gou Young Koh

**Affiliations:** 1 Center for Vascular Research, Institute for Basic Science, Daejeon, South Korea.; 2 Graduate School of Medical Science and Engineering, Korea Advanced Institute of Science and Technology, Daejeon, South Korea.

**Keywords:** Cell biology, Development, Vascular biology, Endothelial cells, Lymph, Transcriptomics

## Abstract

Lymphatics maintain fluid homeostasis, immune surveillance, and tissue integrity. Here, we identified the E26 transformation-specific transcription factors Erg and Fli1 as essential cooperative regulators of lymphatic integrity and function. Using inducible lymphatic endothelial cell–specific deletion in mice, we demonstrated that combined loss of Erg and Fli1 in adults results in fatal lymphatic failure, including chylothorax, chylous ascites, and impaired lymphatic drainage. Single-cell transcriptomic analysis revealed that loss of Erg and Fli1 causes disrupted lymphatic heterogeneity and dysregulation of key lymphatic genes, including valve-specific gene profiles. Erg and Fli1 coordinated lymphatic-immune crosstalk by transcriptionally regulating C-C motif chemokine ligand 21, which mediates DC trafficking. Erg or Fli1 loss also induced proinflammatory and prothrombotic gene expression, further contributing to lymphatic dysfunction. During embryonic development, the codeletion led to lymphatic mispatterning and loss of valve-initiating lymphatic endothelial cell clusters. The impact of loss of Erg and Fli1 function on lymphatic development in mice is consistent with FOXC2 mutations in lymphedema-distichiasis syndrome or *ERG* gene variants underlying primary lymphedema in humans. Moreover, Erg and Fli1 were required for regenerative lymphangiogenesis and lymphatic repair following injury in adults. Our findings establish Erg and Fli1 as core transcriptional regulators of lymphatic identity, integrity, and function.

## Abstract 2

Transcription factors ERG and Fli1 are key regulators that cooperatively maintain lymphatic function and integrity.

## Introduction

Lymphatics play a crucial role in waste clearance, immune surveillance, and maintaining tissue homeostasis throughout the body (1–3). There has been growing interest in understanding the molecular mechanisms that regulate lymphatic vessel development and function, particularly in the context of organ-specific lymphatics (4).

The development and maintenance of lymphatics are orchestrated by a complex interplay of transcription factors and signaling pathways (1–3, 5). Among these, prospero-related homeobox 1 (Prox1) is considered the master regulator of lymphatic endothelial cell (LEC) specification, while vascular endothelial growth factor-C (Vegf-c)/Vegf receptor-3 (Vegfr3) signaling is crucial for lymphangiogenesis and lymphatic vessel integrity (6–9). Additionally, GATA-binding protein 2 (Gata2), forkhead box C2 (Foxc2), and nuclear factor of activated T cells 1 (Nfatc1) work in concert to regulate valve-specific genes in response to fluid shear stress, ensuring proper formation and maturation of lymphatic valves (10–12). Gata2 binds to enhancer element of Prox1, enabling the recruitment of Foxc2 and Nfatc1 to regulate Prox1 transcription (13, 14). Foxc2 acts as a key effector of valve maturation by maintaining lymphatic identity through suppression of blood endothelial genes while promoting expression of valve-specific markers like connexin37 and integrin-α9 (15–17). Nfatc1 mediates the shear stress response by coordinating cytoskeletal remodeling and stabilizing valve-forming endothelial cells (18). These regulatory networks initiate valve development and are crucial for maintaining valve integrity through continuous mechanotransduction.

The transcription factors E26 transformation-specific–related (ETS-related) gene (Erg) and Friend leukemia integration 1 (Fli1), members of the ETS family, have emerged as key regulators of vascular development and homeostasis (19, 20). In blood endothelial cells (BECs), Erg regulates the expression of key vascular genes such as VE-cadherin, von Willebrand factor, and intracellular adhesion molecule-2 for endothelial junction stability and migration (19, 21, 22) and claudin-5 and delta-like canonical notch ligand 4 for angiogenesis and vascular maturation (23, 24). Similarly, Fli1 binds to promoter and enhancer regions of Tie2 and VE-cadherin, which are essential for endothelial cell survival and vascular morphogenesis during the later stages of development (25). Recent studies have highlighted the cooperative activity of Erg and Fli1 for regulating endothelial cell identity and vascular homeostasis. Specifically, their combined deletion in adult endothelial cells resulted in acute multiorgan vascular failure and systemic coagulopathy, leading to death (20, 26).

While the roles of Erg and Fli1 in BECs are relatively well established, their specific functions in LECs remain largely unexplored. Given the shared developmental origin and common features between blood and LECs, Erg and Fli1 may also play crucial roles in lymphatic vessel development and maintenance. Moreover, the unique characteristics, functions, and transcriptional regulations of the lymphatics imply that these transcription factors may have additional or unique roles in the lymphatics. Therefore, in this study, we selectively deleted Erg/Fli1 in LECs in mice. In adult mice, simultaneous deletion of Erg/Fli1 resulted in severe lymphatic dysfunction, leading to chylothorax and chylous ascites, and mortality within 2 weeks. Mechanistically, their codeletion disrupted lymphatic heterogeneity, impaired lymphatic valve identity, and induced a proinflammatory and prothrombotic state. Furthermore, Erg and Fli1 were found to be critical to regenerative lymphangiogenesis and lymphatic recovery following injury. Our findings underscore the cooperative roles of Erg and Fli1 in maintaining lymphatic integrity and function.

## Results

### Erg and Fli1 are critical for lymphatic integrity.

To examine the presence of Erg and Fli1 in lymphatics, we performed immunofluorescence staining in Prox1-GFP lymphatic reporter mice (27). Both Erg and Fli1 are constitutively found in the nuclei of all LECs, consisting of the lymphatics, in the ear skin, lymph node, diaphragm, trachea, and mesentery of adult mice (Supplemental Figure 1, A–D; supplemental material available online with this article; https://doi.org/10.1172/JCI196119DS1).

**Figure 1:**
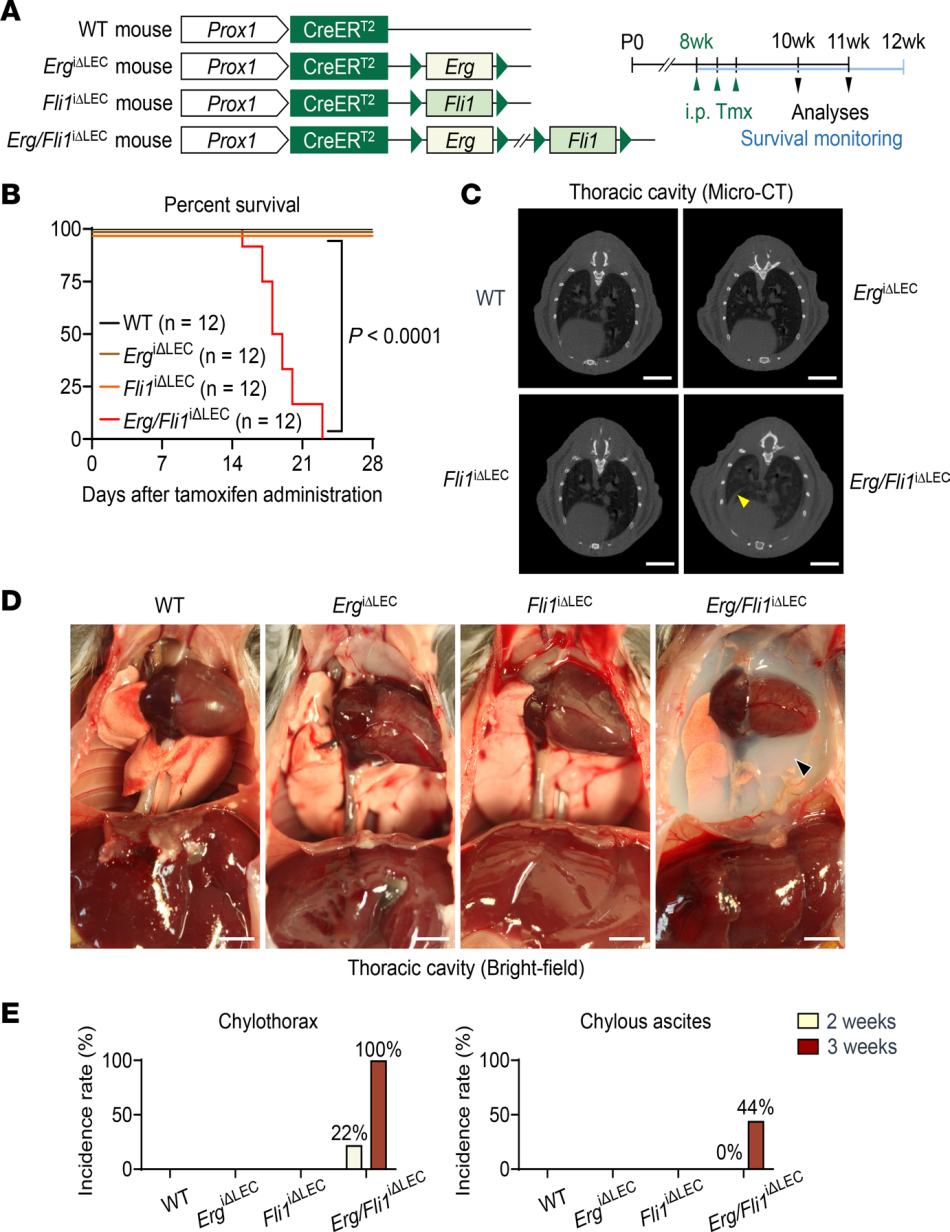
Both Erg and Fli1 are critical for maintaining thoracic duct valve integrity. (**A**) Diagram depicting generation of WT, *Erg*^iΔLEC^, *Fli1*^iΔLEC^, and *Erg/Fli1*^iΔLEC^ mouse lines, i.p. administrations of tamoxifen (Tmx) to adult (8-week-old) mice for 3 consecutive days, and timing for analyses. (**B**) Kaplan-Meier curve showing survival rates of the indicated mice following the initial Tmx administration. *n* = 12 mice/group from 3 independent experiments. *P* value versus WT by Mantel-Cox comparison. (**C** and **D**) Micro-CT images of thoracic cavities and gross bright-field images of the thoraco-abdominal cavity of the indicated mice after 2 weeks of the first Tmx administration. Yellow arrowhead indicates a plural effusion, and black arrowhead indicates chylous fluid. Scale bars: 5 mm. (**E**) Incidence rates of chylothorax and chylous ascites 2 and 3 weeks after the first Tmx administration. *n* = 9 mice/group.

To investigate the role of Erg and Fli1 in maintaining lymphatic integrity, we first generated *Erg*^iΔLEC^ and *Fli1*^iΔLEC^ mice by crossing *Prox1*-Cre-ER^T2^–positive (28) *Erg^fl/+^* mice with *Erg^fl/+^* mice and *Prox1*-Cre-ER^T2^–positive *Fli1^fl/+^* mice with *Fli1^fl/+^* mice (Figure 1A). Cre-ER^T2^–positive but flox/flox-negative mice were used as WT controls among their littermates to exclude a potential toxic effect on cardiovascular tissue induced by tamoxifen-mediated Cre-ER^T2^ activation (29). Additionally, to visualize lymphatic structures more efficiently, we crossed these lines with *Prox1*-GFP mice, generating *Prox1*-GFP-WT (PG-WT), *Prox1*-GFP-*Erg^fl/fl^* (PG-*Erg*^iΔLEC^), and *Prox1*-GFP-*Fli1^fl/fl^* (PG-*Fli1*^iΔLEC^) lines (Supplemental Figure 2A). Using these mutant reporter mice, we confirmed complete LEC-specific deletion of Erg and Fli1 2 weeks after administering tamoxifen per day for 3 consecutive days (Supplemental Figure 2, B–E). However, *Erg*^iΔLEC^ or *Fli1*^iΔLEC^ mice exhibited no noticeable lymphatic abnormalities, such as lymphedema and chylous ascites formation. They showed no reduced survival over a month following the tamoxifen-induced deletion (Figure 1B).

**Figure 2:**
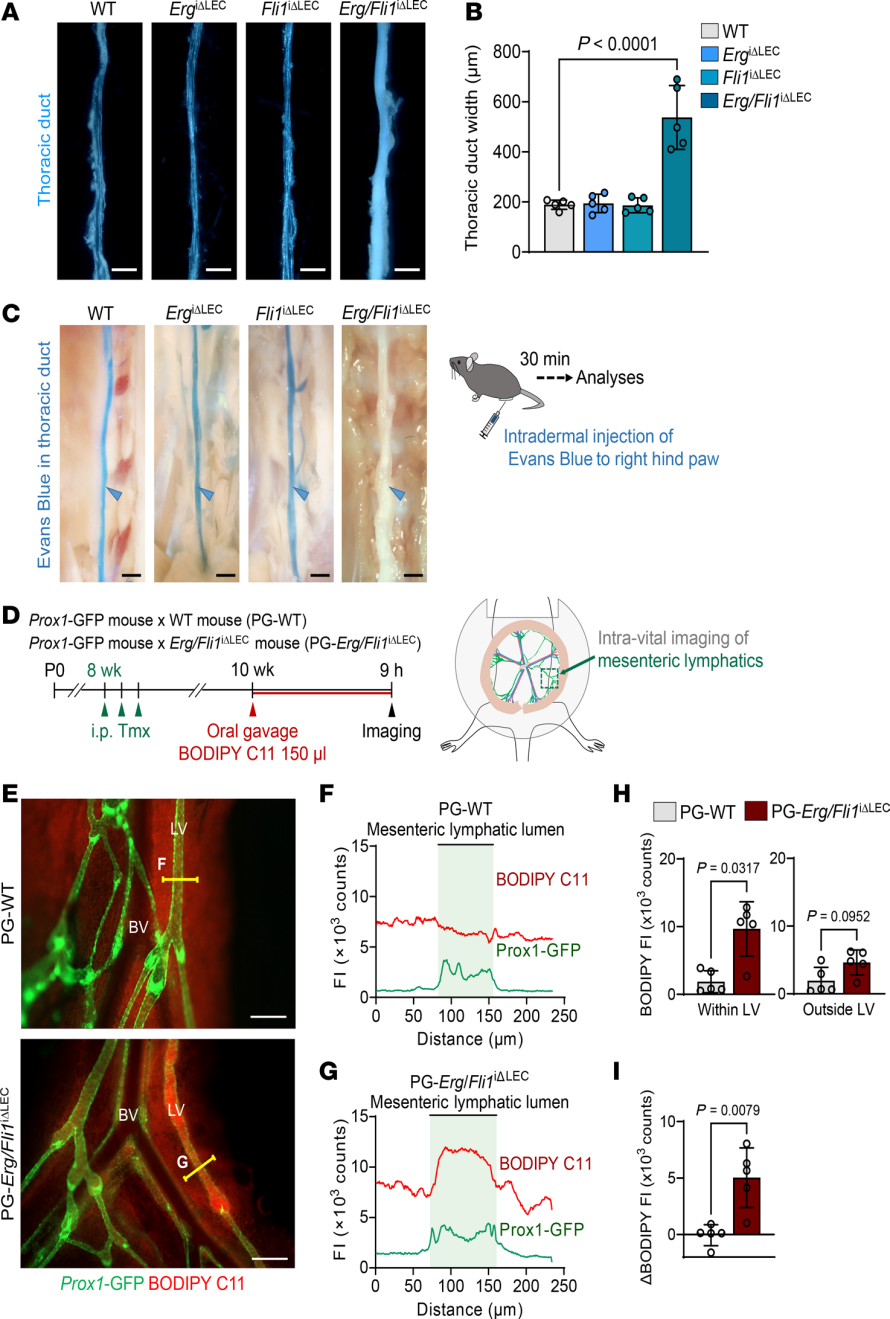
Erg and Fli1 double knockout impairs lymphatic drainage function. (**A** and **B**) Bright-field images and comparison of the width of the thoracic duct at 2 weeks after the first of 3 consecutive days of tamoxifen (Tmx) administrations among the indicated adult mice. Scale bars: 0.5 mm. Each dot indicates a value from 1 mouse, and *n* = 5 mice/group from 2 independent experiments. Data are shown as the mean ± SD; *P* value versus WT by 1-way ANOVA followed by Dunnett’s post hoc test. (**C**) Diagram showing intradermal injection of Evans blue into the right hind paw of the adult mice at 2 weeks after the first of 3 consecutive days of Tmx administration and analysis 30 min later. Bright-field images of the thoracic duct in the indicated mice. Similar findings were obtained from *n* = 5 mice in 3 independent experiments. Scale bars: 0.5 mm. (**D**) Generation of PG-WT and PG-*Erg/Fli1*^i*Δ*LEC^ mice, diagram showing i.p. administrations of Tmx for 3 consecutive days in the adult mice, and 2 weeks later, an intravital imaging of mesenteric lymphatics at 9 h after oral gavage of 150 μL of BODIPY C11. (**E**–**G**) Fluorescence images and fluorescence intensity (FI) profile analysis of BODIPY C11 in the mesenteric lymphatics between PG-WT and PG-*Erg/Fli1*^i*Δ*LEC^ mice at 9 h after the oral gavage. Yellow lines indicate the regions measured for profile analysis. Scale bars: 200 μm. Green shaded regions in the plots represent the mesenteric lymphatic lumens. LV, lymphatic vessel; BV, blood vessel. The letters F and G indicate the regions where BODIPY fluorescence was measured in **F** and **G**. (**H** and **I**) Comparisons of mean and retained BODIPY C11 FI in the mesenteric lymphatics of PG-WT and PG-*Erg/Fli1*^i*Δ*LEC^ mice. Mean FIs of 3 regions within the lymphatics (Within LV) or adjacent nonlymphatic regions (Outside LV) are shown. Retained BODIPY C11 FI (ΔBODIPY FI) was defined as FI_Within_ _LV_ – FI_Outside_ _LV_. Each dot indicates a value from 1 mouse, and *n* = 5 mice/group from 2 independent experiments. Data are shown as the mean ± SD; *P* value versus WT by 2-tailed Mann-Whitney *U* test.

To address the potential redundant roles of Erg and Fli1 in maintaining lymphatic integrity, we generated *Erg/Fli1*^iΔLEC^ double deletion mice by crossing *Prox1*-Cre-ER^T2^–positive *Erg^fl/+^/Fli1^fl/+^* mice with *Erg^fl/+^/Fli1^fl/+^* mice (Figure 1A). We also generated PG-*Erg/Fli1*^iΔLEC^ mice by crossing *Prox1*-GFP mice with *Erg/Fli1*^iΔLEC^ mice for efficient lymphatic structure visualization (Supplemental Figure 2A). In these mutant mice, we confirmed the specific and complete deletion of both Erg and Fli1 in LEC 2 weeks after administering tamoxifen per day for 3 consecutive days (Supplemental Figure 2, F and G). Surprisingly, most *Erg/Fli1*^iΔLEC^ mice died within a month following the tamoxifen-induced deletion, primarily due to severe chylothorax and the formation of chylous ascites, as evidenced by micro-CT imaging and autopsy analyses (Figure 1, B–E). Compared with WT mice, *Erg/Fli1*^iΔLEC^ mice exhibited a 2.8-fold increase in the width of the thoracic duct, accompanied by a marked amount of chylous coagulant (Figure 2, A and B). In contrast, no differences were observed in the *Erg*^iΔLEC^ or *Fli1*^iΔLEC^ mice. The Evans blue drainage assay, which assesses lymph flow through the thoracic duct, confirmed these findings; no drainage was observed in *Erg/Fli1*^iΔLEC^ mice (Figure 2C). To assess and compare the drainage functions of mesenteric lymphatics, we performed an oral gavage loading assay using BODIPY C11–labeled fatty acids (30) in PG-WT and PG-*Erg/Fli1*^iΔLEC^ mice (Figure 2D). We observed a marked stasis of the drained tracer within the mesenteric lymphatics in the PG-*Erg/Fli1*^iΔLEC^ mice, whereas PG-WT mice showed normal clearance (Figure 2, E–I). Furthermore, compared with WT mice, the *Erg/Fli1*^iΔLEC^ mice exhibited significant impairments in lymphatic drainage from the inguinal lymph node to the ascending axillary lymph node via dermal lymphatics in the thoracoabdominal cavity as well as from the foot pad to the popliteal and inguinal lymph nodes (Supplemental Figure 3, A–G). Thus, Erg and Fli1 are critical for lymphatic function.

To examine whether the Erg/Fli1 deletion in lymphatics affects the functional integrity of neighboring blood vessels, we compared blood vascular permeability in the mesentery between WT and *Erg/Fli1*^iΔLEC^ mice using an intravenous injection of Evans blue dye (Supplemental Figure 4A). No significant difference in vascular leakage in the mesentery between the 2 groups was found over 1 h after the Evans blue injection (Supplemental Figure 4, B and C), indicating that the Erg/Fli1 deletion in lymphatics does not notably compromise surrounding blood vascular function.

### Lymphatic heterogeneity is disrupted in Erg/Fli1^iΔLEC^ mice.

**Figure 3:**
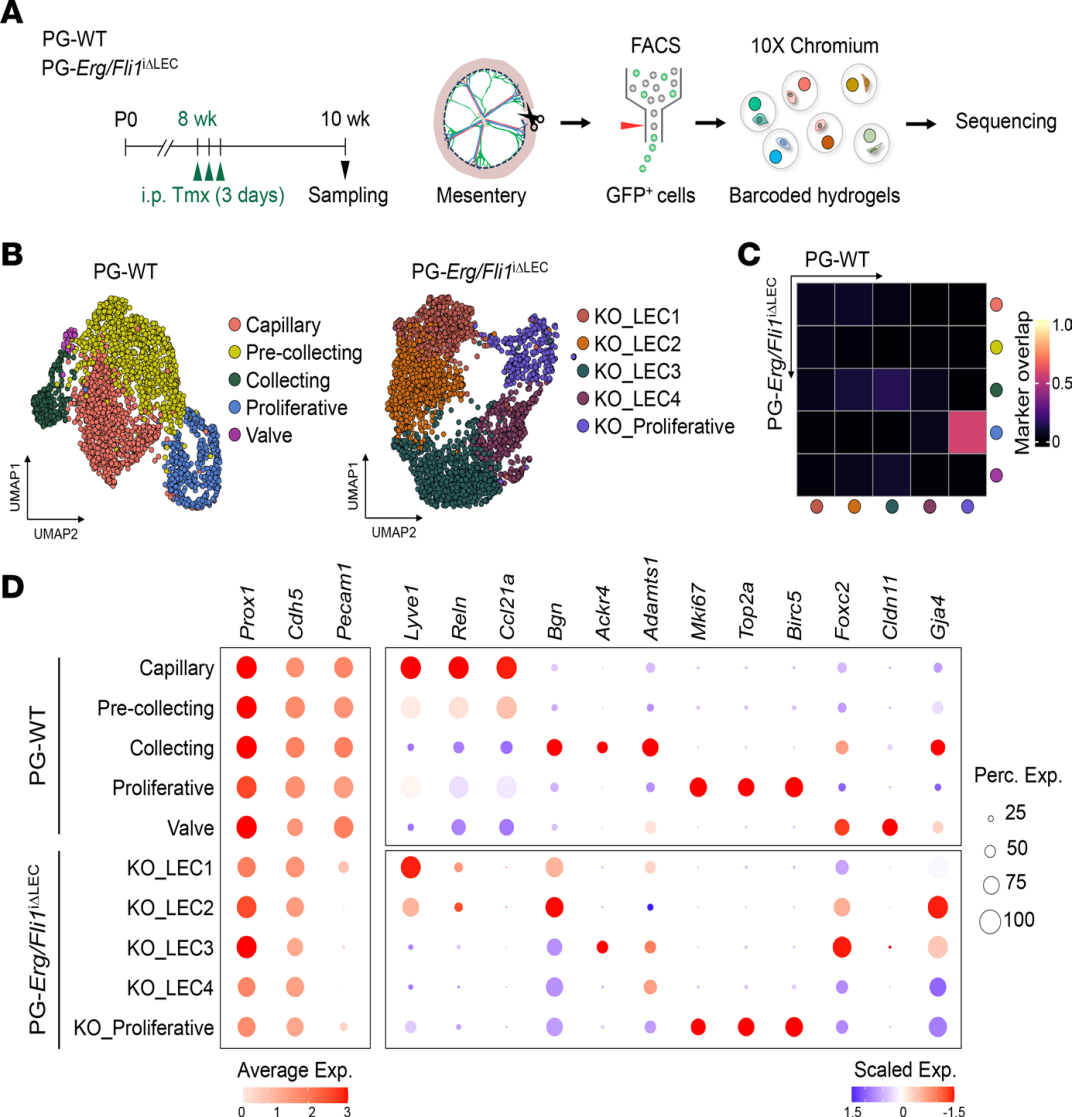
Loss of lymphatic heterogeneity in *Erg/Fli1*^iΔLEC^ mice. (**A**)Diagram showing i.p. administrations of tamoxifen (Tmx) for 3 consecutive days to PG-WT and PG-*Erg/Fli1*^iΔLEC^ mice, and sampling and scRNA-seq of mesenteric LECs. (**B**) UMAP visualization of mesenteric LEC subtypes in PG-WT and PG-*Erg/Fli1*^iΔLEC^ mice datasets. (**C**) Heatmap showing Jaccard similarities between top 100 marker genes for each cluster from PG-WT and PG-*Erg/Fli1*^iΔLEC^ mice datasets. (**D**) Dot plots visualizing average expression levels of *Prox1*, *Cdh5*, and *Pecam1* (left panel) per cluster and scaled expression levels of marker genes for LEC subtypes (right panel) in PG-WT and PG-*Erg/Fli1*^iΔLEC^ mice datasets. Scaled expression levels are scored across LEC subtypes in each dataset.

To gain insights into the transcriptomic changes associated with these pathological phenotypes, we conducted scRNA-seq analysis on LECs isolated from the mesenteric lymphatics of PG-WT and PG-*Erg/Fli1*^iΔLEC^ mice (Figure 3A). The GFP-labeled LECs were isolated and enriched from the lymphatics in the entire mesentery that was dissected along the intestine (see details in Methods) using flow cytometry. Subsequently, we performed droplet-based scRNA-seq utilizing the 10x Genomics platform (Figure 3A). Unsupervised clustering of mesenteric LECs from PG-WT mice identified distinct lymphatic subtypes with unique transcriptomic profiles (Figure 3B and Supplemental Figure 5A). Five LEC subtypes were annotated based on the specific expression of certain signatures (15, 31), which are as follows: capillary (*Lyve1*^+^, *Reln*^+^, *Ccl21a*^+^), collecting (*Lyve1*^–^, *Ccl21a*^–^, *Bgn*^+^), valve (*Foxc2*^+^, *Cldn11*^+^), proliferative (*Mki67^+^*, *Top2a^+^*, *Birc5*^+^), and precollecting (showing intermediate expression of both capillary and collecting signatures) (Figure 3D). Unsupervised clustering of mesenteric lymphatics derived from the PG-*Erg/Fli1*^iΔLEC^ dataset revealed distinct subpopulations (Supplemental Figure 5B). We used the top 100 differential markers that distinguish lymphatic subtypes in the WT dataset as a reference to assess the correlations among the clusters between datasets. We then computed Jaccard similarities with the markers for the clusters in the PG-*Erg/Fli1*^iΔLEC^ dataset. With the exception of the cluster identified as proliferative, all other clusters in the PG-*Erg/Fli1*^iΔLEC^ dataset demonstrated low marker correlations (Figure 3C). Additionally, expression of lymphatic subtype-specific markers indicative of capillary, collecting, and valve identities was not observed in the PG-*Erg/Fli1*^iΔLEC^ dataset (Figure 3D).

**Figure 4:**
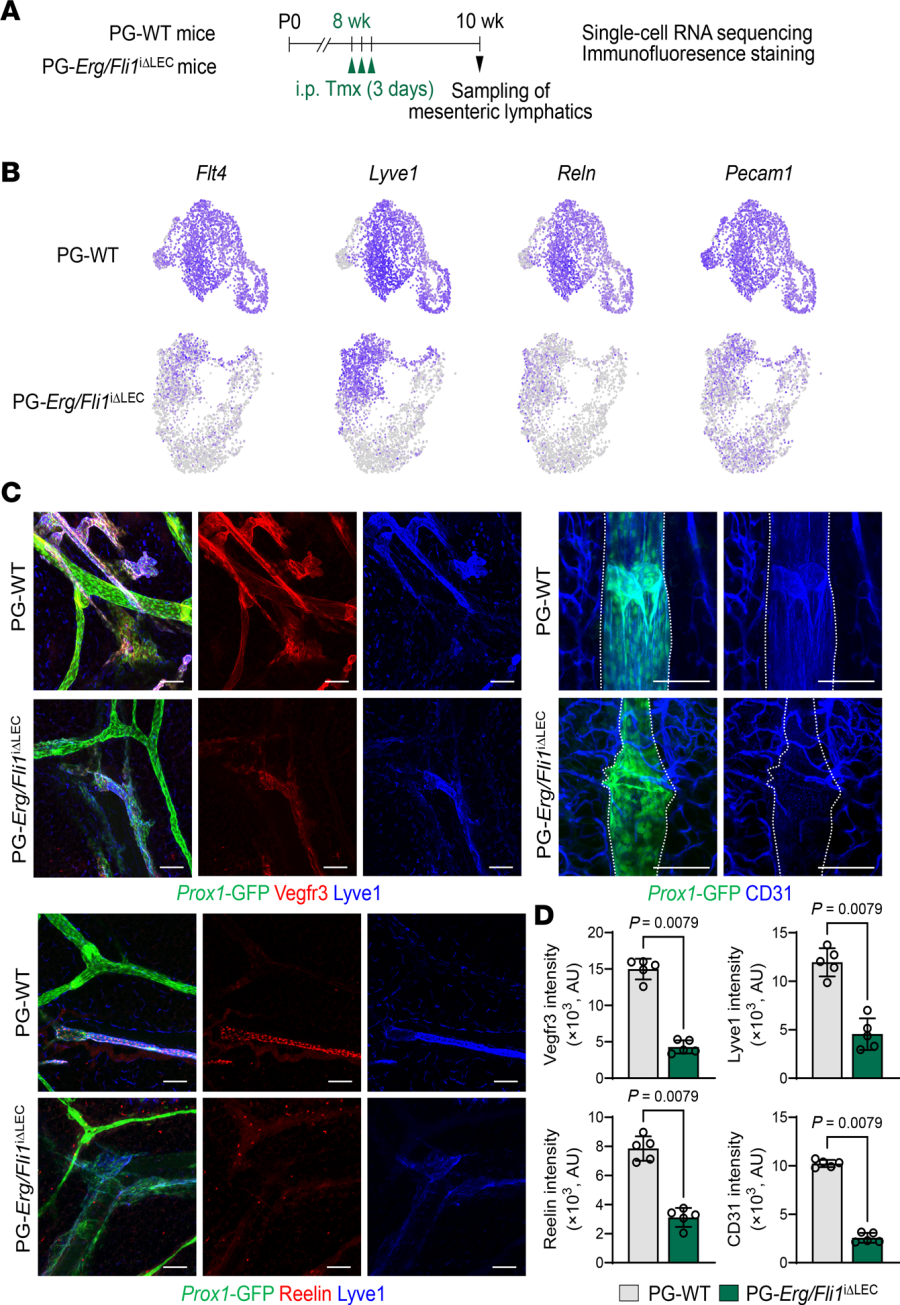
Lymphatic signature genes and proteins are downregulated in the lymphatics of *Erg/Fli1*^iΔLEC^ mice. (**A**) Diagram showing i.p. administrations of tamoxifen (Tmx) for 3 consecutive days to PG-WT and PG-*Erg/Fli1*^iΔLEC^ mice and sampling of mesenteric lymphatics for scRNA-seq and immunofluorescence staining. (**B**) UMAP plots visualizing mRNA expression levels of *Flt4*, *Lyve1*, *Reln,* and *Pecam1* in PG-WT and PG-*Erg/Fli1*^iΔLEC^ mice datasets. (**C** and **D**) Immunofluorescence images and comparisons of protein levels of Vegfr3, Lyve1, Reelin, and CD31 (PECAM1) in the mesenteric lymphatics between PG-WT and PG-*Erg/Fli1*^iΔLEC^ mice at 2 weeks after the first of 3 consecutive days of Tmx administration. Lymphatic vessels are marked with white dashed lines based on *Prox1*-GFP^+^ cells. Scale bars: 100 μm. Each dot indicates a value from 1 mouse, and *n* = 4–5 mice/group from 2 independent experiments. Data are shown as the mean ± SD; *P* value versus WT by 2-tailed Mann-Whitney *U* test.

Notably, compared with the mesenteric LECs in PG-WT mice, those in PG-*Erg/Fli1*^iΔLEC^ mice exhibited marked reductions in the expression of key lymphatic genes, including *Flt4*, *Lyve1*, *Reln*, and *Pecam1* (32, 33) (Figure 3D and Figure 4, A and B). We confirmed these changes through immunofluorescence staining of lymphatics in the mesentery and ear skin. Compared with WT, mesenteric lymphatics of PG-*Erg/Fli1*^iΔLEC^ mice demonstrated a marked reduction of Vegfr3 (71.3%), Lyve1 (62%), reelin (60.3%), and CD31 (74.9%) (Figure 4, C and D). Consistently, lymphatics in the ear skin of PG-*Erg/Fli1*^iΔLEC^ mice showed significantly reduced CD31 (65.8%), Vegfr3 (87.6%), Lyve1 (16.4%), and reelin (86.4%) compared with PG-WT mice (Supplemental Figure 6, A and B). Thus, our clustering analysis revealed that the codeletion of Erg and Fli1 disrupts lymphatic heterogeneity and identity.

Given the broad developmental importance of Erg and Fli1 in specialized valve programs across vascular systems, including the cardiac valve (34), we assessed the expression and roles of Erg and Fli1 in the cardiac valvular cells. In the mitral valves of PG-WT mice, we confirmed coexpression of Erg and Fli1 in Prox1^+^ valvular cells, where PG-*Erg/Fli1*^iΔLEC^ mice showed marked deletions of Erg and Fli1 (Supplemental Figure 7, A and B). Interestingly, Prox1^+^ lymphatics were present in the leaflet surface near the annulus area of the mitral valve (Supplemental Figure 7A).

Despite these deletions over 10–14 days, histological analysis showed similar valve morphology, and echocardiography revealed no significant differences in ejection fraction, fractional shortening, stroke volume, or left ventricular mass between the 2 groups (Supplemental Figure 8, A–C). These data suggest that although PG-*Erg/Fli1*^iΔLEC^ deletes Erg and Fli1 in Prox1^+^ cardiac valve cells, these deletions over a short period do not impair cardiac valve structure or function in adult mice. However, further examination is needed to determine whether a longer period of deletions could lead to potential structural and functional changes in cardiac valves in the future.

### Erg and Fli1 positively regulate Ccl21 expression in lymphatics.

**Figure 5:**
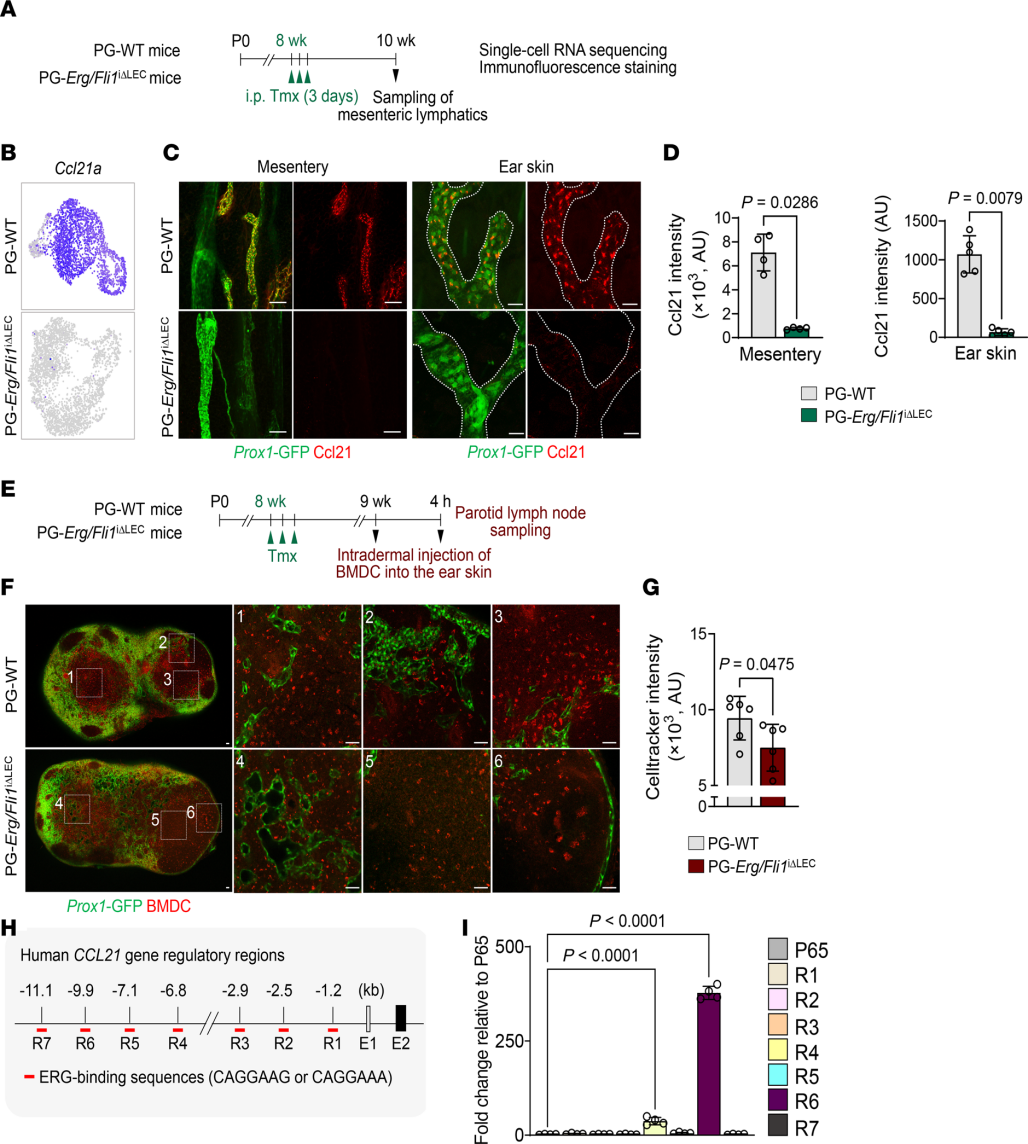
*Ccl21* is regulated by Erg and Fli1 in LECs. (**A**) Diagram showing i.p. administrations of tamoxifen (Tmx) for 3 consecutive days to PG-WT and PG-*Erg/Fli1*^iΔLEC^ mice, and sampling of mesenteric lymphatics for scRNA-seq and immunofluorescence staining. (**B**) UMAP plots visualizing expression levels of *Ccl21a* in PG-WT and PG-*Erg/Fli1*^iΔLEC^ datasets. (**C** and **D**) Immunofluorescence images and comparisons of ear skin lymphatics and their Ccl21 protein levels in PG-WT and PG-*Erg/Fli1*^iΔLEC^ mice at 2 weeks after the first of 3 consecutive days of Tmx administration. Scale bars: 50 μm. White dotted lines outline the ear skin lymphatics. Each dot indicates a value from 1 mouse, and *n* = 5 mice/group from 2 independent experiments. Data are shown as the mean ± SD; *P* value versus WT by 2-tailed Mann-Whitney *U* test. (**E**) Diagram showing the schedule of i.p. administrations of Tmx for 3 consecutive days, and then 1 week later, intradermal injection of CellTracker-labeled BMDCs into the ear skin followed by the ipsilateral parotid lymph node sampling 4 h later in PG-WT and PG-*Erg/Fli1*^iΔLEC^ mice. (**F** and **G**) Fluorescence images and comparison of fluorescence intensity of CellTracker-labeled BMDCs in the parotid lymph node of the indicated mouse line. Scale bars: 50 μm. Each dot indicates a value from 1 mouse, and *n* = 6 mice/group from 2 independent experiments. *P* values versus WT by 2-tailed Welch’s *t* test. (**H** and **I**) ChIP analysis in primary cultured human LECs with anti-ERG antibody followed by qPCR using primers specific for the indicated regions of the *CCL21* gene depicted in R1–R7 (**H**) demonstrates ERG interacts with *CCL21* gene regulatory regions located at both –6.8 and −9.9 kb upstream of the transcription start site of the *CCL21* gene (**I**). The sequence of the ERG binding motif is limited to CAGGAAG or CAGGAAA for qPCR. *RELA* (*P65*) was used as a negative control in ChIP-qPCR. Each dot indicates a value from 1 sample, and *n* = 4 samples/group from 2 independent experiments. Data are shown as the mean ± SD; *P* value versus WT by 1-way ANOVA followed by Dunnett’s post hoc test.

Among the reduced genes, we focused on C-C motif chemokine ligand 21 (Ccl21), a chemokine uniquely expressed in the capillary LECs. Ccl21 production at the distal ends of lymphatic vessels establishes a chemokine gradient essential for T cell and DC trafficking into lymphatics and toward draining lymph nodes (35–38). Despite its central role in immunity, the upstream genetic regulation of Ccl21 remains unclear. Strikingly, our analyses of scRNA differential testing and immunostaining indicated that both *Ccl21a* mRNA expression and Ccl21 protein were almost absent in the LECs of PG-*Erg/Fli1*^iΔLEC^ mice at 2 weeks after the first tamoxifen injection (Figure 5, A–D). This significant reduction of Ccl21 protein was observed in mesenteric and ear skin lymphatics as soon as 4 days after the deletion (Supplemental Figure 9, A–E). In addition, RT-PCR analysis confirmed the reduction of *CCL21* mRNA in human LECs at 48 h after ERG and FLI1 depletion (Supplemental Figure 9, F and G). These data imply that Erg and Fli1 directly and positively regulate *Ccl21* expression in LECs.

To assess the functional consequence of Ccl21 deficiency in Erg/Fli1-deficient lymphatics, we performed a DC migration assay according to a previous report (37). Bone marrow–derived dendritic cells (BMDCs), stimulated with lipopolysaccharide and labeled with CellTracker Red CMTPX, were intradermally injected into the ear skin of PG-WT or PG-*Erg/Fli1*^iΔLEC^ mice. Four hours after injection, compared with PG-WT, the parotid lymph nodes of PG-*Erg/Fli1*^iΔLEC^ mice exhibited a significant reduction (20.6%) in the fluorescence intensity of labeled BMDCs (Figure 5, E–G).

To determine whether ERG directly regulates *CCL21* expression, we conducted ChIP-qPCR using primary cultured human LECs. We used primers targeting the ERG-binding motif on the *CCL21* promoter and its upstream gene regulatory regions (Supplemental Table 1). The ChIP-qPCR analysis revealed that endogenous ERG bound to 2 specific regions, designated as R4 and R6, which are located 6.8 and 9.9 kb upstream of the *CCL21* transcription start site in human LEC, respectively. Notably, R6 displayed a stronger binding affinity for ERG than R4, with binding efficiencies of 377.9- and 37.3-fold relative to the control *RELA*, respectively (Figure 5, H and I). These findings indicate that ERG directly and positively regulates *CCL21* expression in human LECs.

### Erg and Fli1 regulate genes essential for maintaining lymphatic valve integrity.

**Figure 6:**
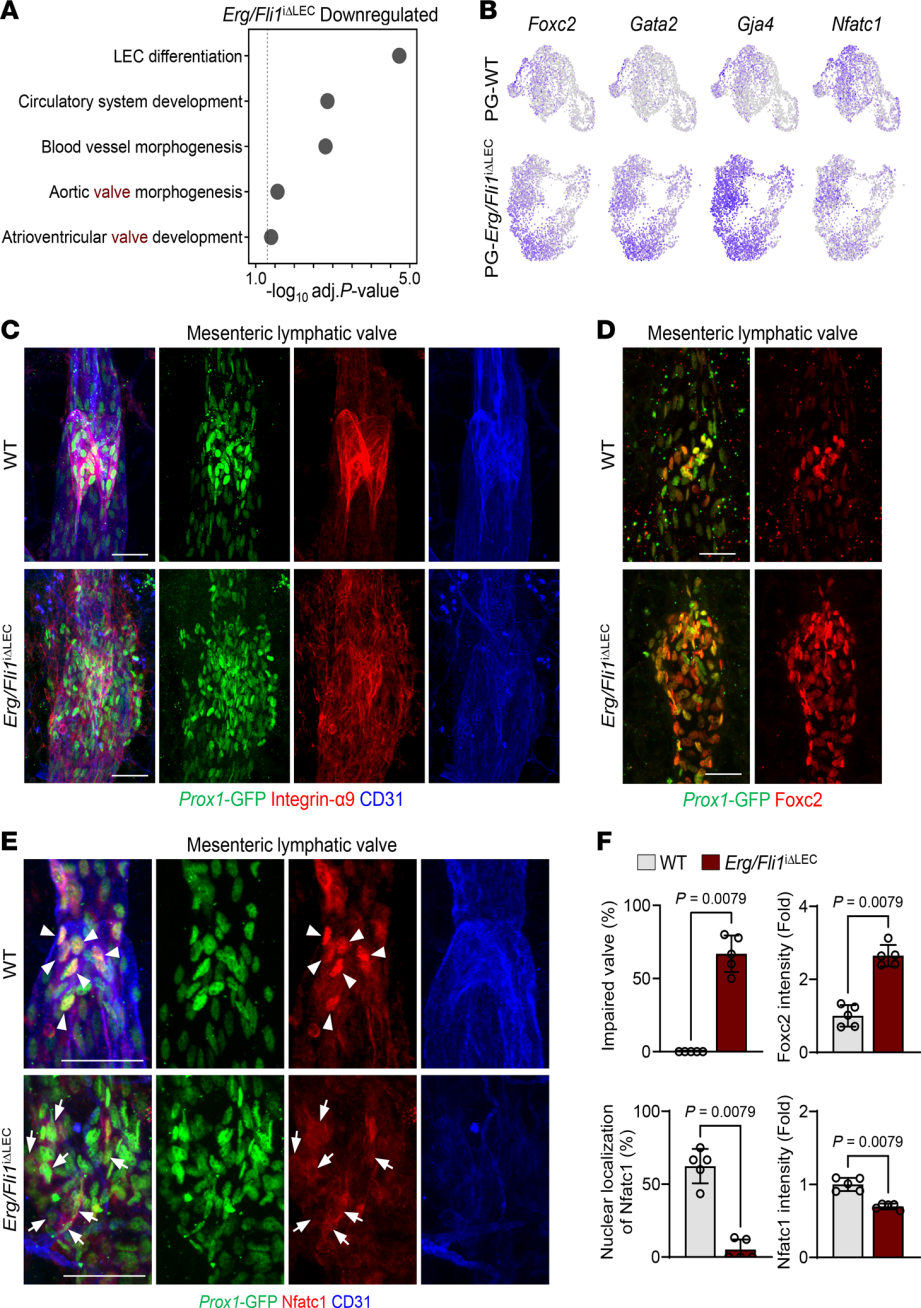
Dysregulation of lymphatic valve markers in *Erg/Fli1*^iΔLEC^ mice. (**A**–**F**) Diagram showing i.p. administrations of tamoxifen (Tmx) for 3 consecutive days to PG-WT and PG-*Erg/Fli1*^iΔLEC^ mice or WT and *Erg/Fli1*^iΔLEC^ mice, and sampling of mesenteric lymphatics at 2 weeks after the first Tmx injection for scRNA-seq and immunofluorescence staining. (**A**) Dot plots showing significantly enriched GO terms of top 100 downregulated genes in PG-*Erg/Fli1*^iΔLEC^ LECs compared with PG-WT LECs. Selected terms with Benjamini-Hochberg method–adjusted *P* value < 0.05 are shown. (**B**) UMAP plots visualizing expression levels of *Foxc2*, *Gata2*, *Gja4*, and *Nfatc1* in PG-WT and PG-*Erg/Fli1*^iΔLEC^ datasets. (**C**–**E**) Immunofluorescence images of Prox1^hi^Integrin-α9^hi^CD31^hi^ mesenteric lymphatic valves, Prox1^hi^Foxc2^hi^ mesenteric lymphatic valves, and Prox1^hi^Nfatc1^hi^ in mesenteric lymphatic valves in WT and *Erg/Fli1*^iΔLEC^ mice. Arrowheads indicate nuclear localization of Nfatc1, and arrows indicate cytoplasmic localization of Nfatc1. Similar findings are shown from *n* = 5 mice/group from 2 independent experiments. Scale bars: 50 μm. (**F**) Comparisons of impaired valves, Foxc2 intensity, nuclear localization of Nfatc1, and Nfatc1 intensity in mesenteric lymphatic valve areas between WT and *Erg/Fli1*^iΔLEC^ mice. Each dot indicates a value from 1 mouse, and *n* = 5 mice/group from 2 independent experiments. Data are shown as the mean ± SD; *P* value versus WT by 2-tailed Mann-Whitney *U* test.

To further explore the transcriptomic changes in the mesenteric lymphatics of PG-*Erg/Fli1*^iΔLEC^ mice, we performed Gene Ontology (GO) term analysis on the top 100 downregulated genes from pseudo-bulk transcriptomic comparisons of LECs between PG-WT and PG-*Erg/Fli1*^iΔLEC^ mice single-cell datasets (Figure 6A). This analysis revealed significant enrichment in GO terms related to lymph endothelial cell differentiation, circulatory system development, blood vessel morphogenesis, and processes associated with aortic valve and atrioventricular valve development (Figure 6A). These findings underscore the essential role of Erg/Fli1 in regulating both blood vascular and lymphatic development, including the formation and function of valves. After observing defects in lymphatic drainage, we investigated the integrity of lymphatic valves in the *Erg/Fli1*^iΔLEC^ mice. Our transcriptomic analysis showed that key lymphatic valve signatures, such as *Foxc2*, *Gata2*, *Gja4*, and *Nfatc1*, were selectively expressed within the valve cluster of PG-WT mice (Figure 6B). In contrast, these signatures were broadly upregulated across all LEC clusters in PG-*Erg/Fli1*^iΔLEC^ mice, except for *Nfatc1*, which showed reduced expression (Figure 6B), indicating a disrupted transcriptional regulation of valve identity.

The typical semiluminal shape of the integrin-α9^hi^ and Foxc2^hi^ lymphatic valves was markedly altered in the *Erg/Fli1*^iΔLEC^ mice compared with the WT mice (Figure 6C). Notably, Foxc2^hi^ LECs, which are restricted to valve regions in WT mice, were upregulated (2.65-fold of WT) and observed throughout the lymphangion of *Erg/Fli1*^iΔLEC^ mice. Additionally, Nfatc1 showed reduced expression (0.7-fold of WT) and a shift from nuclear localization to cytoplasmic localization in the LECs of the mesenteric lymphatics in *Erg/Fli1*^iΔLEC^ mice (Figure 6, D–F). To further investigate the cellular dynamics within lymphatic valves following Erg/Fli1 deletion, we administered EdU 1 week after the deletion and analyzed the mice a week later (Supplemental Figure 10A). In the mesenteric lymphatics, we observed a significant increase in Prox1^+^ EdU^+^ LECs in PG-*Erg/Fli1*^iΔLE^ mice compared with PG-WT mice, indicating abnormal hyperproliferation after Erg/Fli1 deletion (Supplemental Figure 10, B and D). In contrast, there were no significant changes in the number of Prox1^+^ caspase-3^+^ LECs in both groups (Supplemental Figure 10, C and D). These results suggest that Erg/Fli1 deletion disrupts lymphatic valve integrity partially by abnormal hyperproliferation of valve LECs, rather than through valvular cell loss.

These observations led us to investigate whether ERG and FLI1 directly regulate these genes at the transcriptional level in the primary cultured human dermal LECs. We selectively depleted ERG, FLI1, or both ERG and FLI1 in the LECs using specific siRNA (see details in Methods). When compared with the control siRNA, the simultaneous knockdown of both ERG and FLI1 using cotransfection of ERG siRNA and FLI1 siRNA resulted in significant reductions in VEGFR3 (0.7-fold) and NFATC1 (0.49-fold) and increases in GATA2 (2.1-fold) and FOXC2 (1.4-fold) compared with control siRNA (Supplemental Figure 11, A and B). Thus, Erg/Fli1 is crucial in maintaining lymphatic valve integrity through cooperative activities.

### Erg and Fli1 negatively regulate the genes related to inflammation and coagulation in LEC.

**Figure 7:**
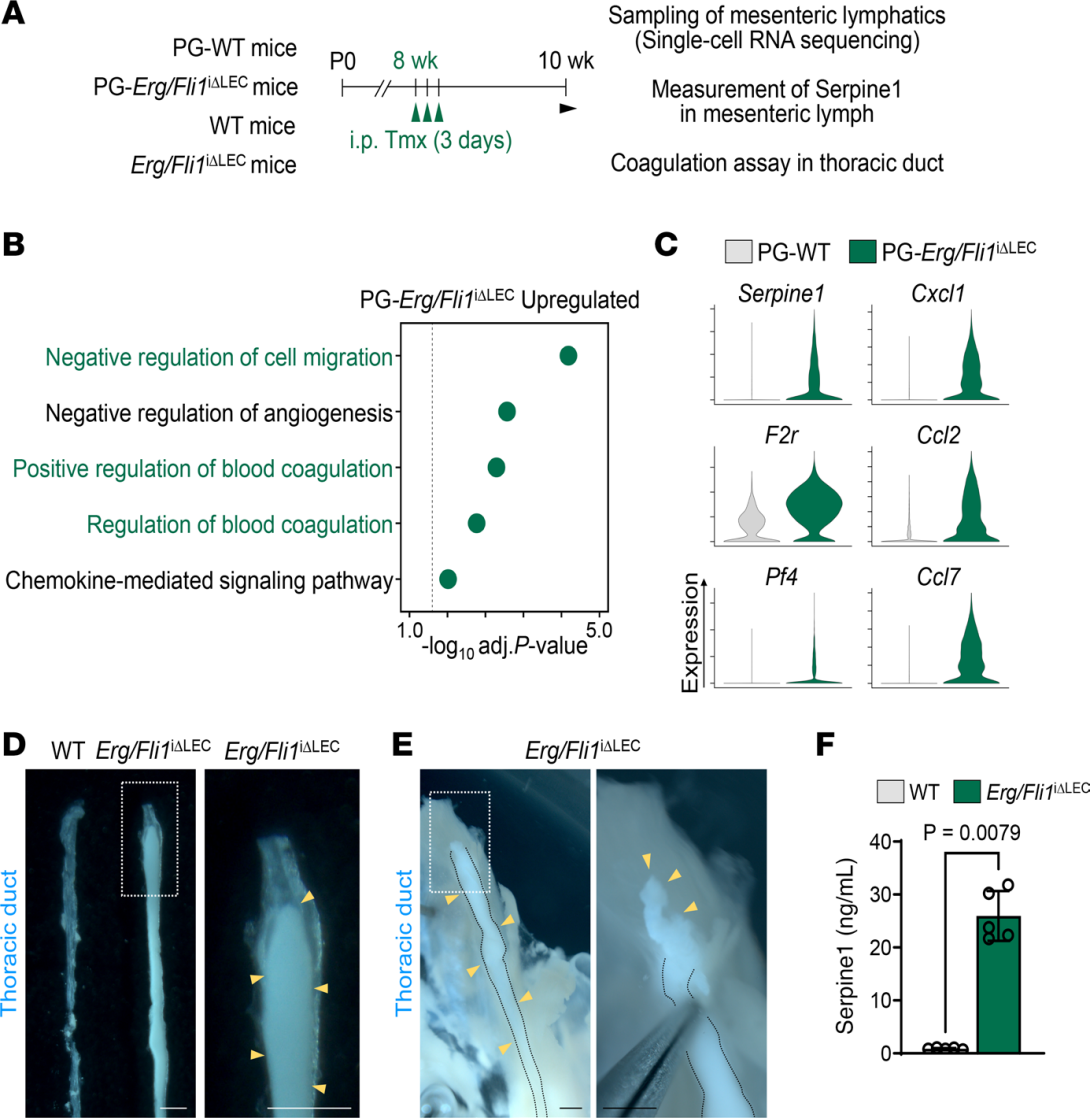
Upregulation of pro-coagulation factors and chemokines in *Erg/Fli1*^iΔLEC^ mice. (**A**) Diagram showing i.p. administrations of tamoxifen (Tmx) for 3 consecutive days to PG-WT and PG-*Erg/Fli1*^iΔLEC^ mice or WT and *Erg/Fli1*^iΔLEC^ mice, and sampling of mesenteric lymphatics for scRNA-seq, sampling of mesenteric lymph for measuring Serpine1, and coagulation assay in thoracic duct at 2 weeks after the first Tmx injection. (**B**) Dot plots showing significantly enriched GO terms of top 100 upregulated genes in PG-*Erg/Fli1*^iΔLEC^ LECs compared with PG-WT LECs. Selected terms with Benjamini-Hochberg method-adjusted *P* value < 0.05 are shown. (**C**) Violin plots showing expression levels of genes related to coagulation (*Serpine1*, *F2r*, and *Pf4*) and chemokines (*Cxcl1*, *Ccl2*, and *Ccl7*) in PG-WT and PG-*Erg/Fli1*^iΔLEC^ LECs. (**D** and **E**) Images of thoracic ducts in WT and *Erg/Fli1*^iΔLEC^ mice. White dashed boxes are magnified in the right panels. Black dashed lines outline thoracic ducts, and yellow arrowheads indicate coagulated lymph chyle. Scale bars: 500 μm. (**F**) Comparison of protein levels of Serpine1 in the mesenteric lymph of WT and *Erg/Fli1*^iΔLEC^ mice. Each dot indicates a value from 1 mouse, and *n* = 5 mice/group from 2 independent experiments. Data are shown as the mean ± SD; *P* value versus WT by 2-tailed Mann-Whitney *U* test.

Further analysis showed that the GO term analysis of the top 100 upregulated genes in the PG-*Erg/Fli1*^iΔLEC^ mesenteric lymphatics highlighted pathways related to cell migration, coagulation, and chemokine-mediated signaling (Figure 7, A and B). Notably, we observed a marked increase in the expression of prothrombotic factors, including *Serpine1*, *F2r*, and *Pf4* (Figure 7C) (39, 40). Consistent with these molecular findings, substantial coagulation of a gel-like chyle was evident in the thoracic duct when it was compressed with forceps (Figure 7, D and E). Given the established role of Serpine1 as a key mediator of thrombosis through inhibiting fibrinolysis (40), we quantified the protein levels from the lymph directly collected from the mesenteric lymphatics. Consistent with the transcriptomic findings, lymph from *Erg/Fli1*^iΔLEC^ mice exhibited a 30.5-fold elevation in Serpine1 level compared with WT controls (Figure 7F). Additionally, chemokine genes *Cxcl1*, *Cxcl2*, and *Ccl7* were upregulated (Figure 7C). Thus, *Erg/Fli1*^iΔLEC^ mesenteric lymphatics are prothrombotic and proinflammatory.

To identify transcriptional programs regulated by Erg/Fli1 that are specific to lymphatic, but not in BECs, we compared differentially expressed genes (DEGs) identified in our dataset with those reported in a recent study analyzing BECs after Erg/Fli1 deletion (20) (Supplemental Figure 12A). We first examined genes downregulated in either LECs or BECs following Erg/Fli1 loss. In line with our observations, LECs selectively downregulated genes associated with lymphatic fate commitment, leukocyte migration, and the Ccl21 signaling pathway (Supplemental Figure 12B). In contrast, proinflammatory chemokine genes such as *Ccl2*, *Ccl7*, and *Cxcl2* were specifically upregulated in LECs, accounting for the proinflammatory phenotype following Erg/Fli1 deletion. Given that Erg/Fli1 are key transcription factors for endothelial identity, numerous genes were commonly dysregulated in both LECs and BECs. Consistent with previous findings, Erg/Fli1 deletion upregulated genes involved in coagulation, including *Serpine1*, *F2r*, and *Procr* (Supplemental Figure 12C). Additionally, genes associated with angiogenesis as well as endothelial cell chemotaxis and migration were downregulated in both cell types (Supplemental Figure 12D). Together, these results demonstrate that Erg/Fli1 orchestrates core transcriptional networks essential for both vascular systems while also governing lymphatic-specific transcriptional programs that are not shared with BECs.

### Erg and Fli1 are critical for lymphatic plexus patterning during embryonic development.

**Figure 8:**
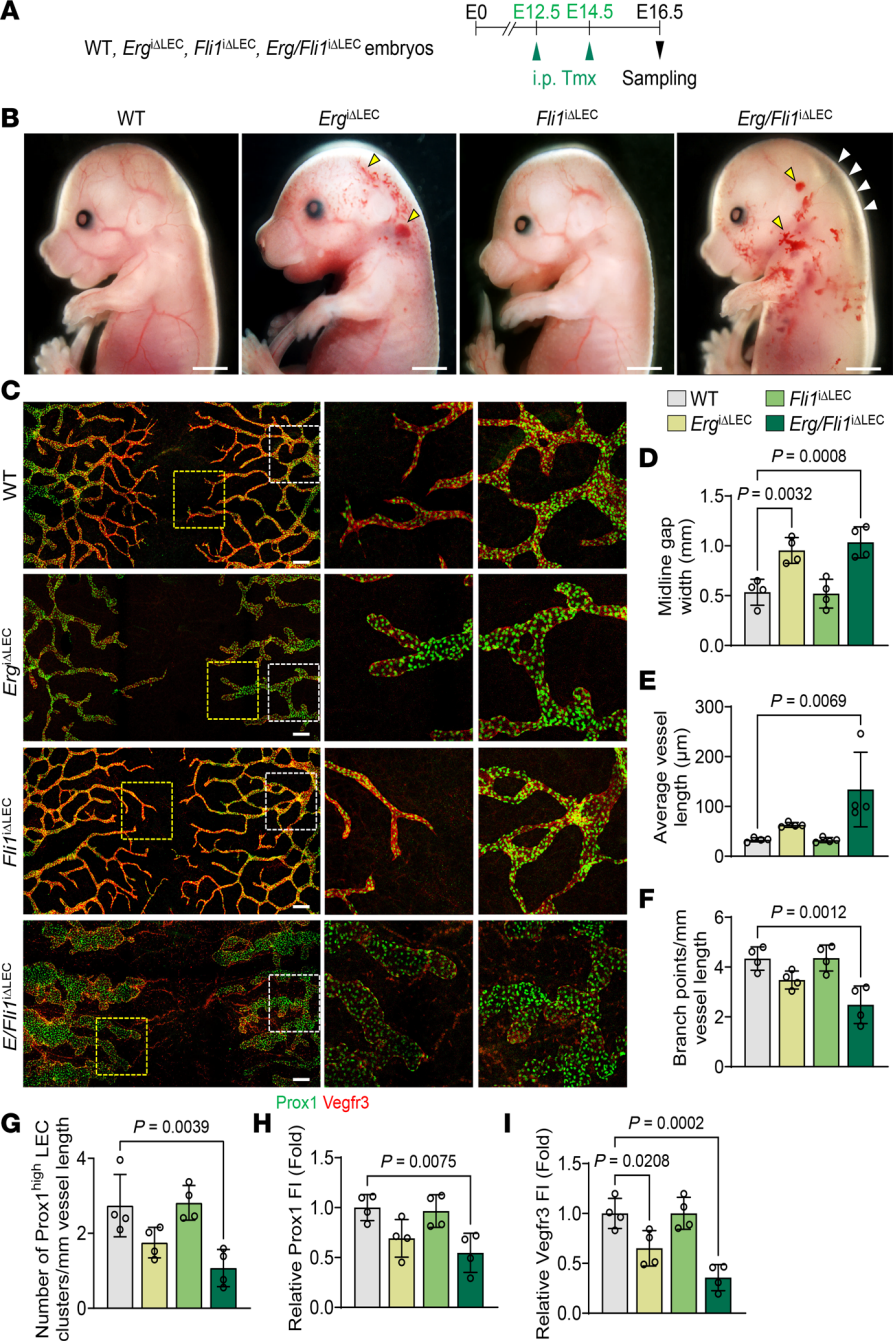
Erg and Fli1 are essential for lymphatic plexus patterning during embryonic development. (**A**) Diagram depicting tamoxifen (Tmx) administrations to WT, *Erg*^iΔLEC^, *Fli1*^iΔLEC^, and *Erg/Fli1*^iΔLEC^ embryos at E12.5 and E14.5 and analyses at E16.5. (**B**) Images of WT, *Erg*^iΔLEC^, *Fli1*^iΔLEC^, and *Erg/Fli1*^iΔLEC^ embryos at E16.5. White and yellow arrowheads indicate hemorrhage and edema. Scale bars: 2 mm. (**C**–**I**) Immunofluorescence images of Prox1 and Vegfr3 expression in E16.5 dorsal skin lymphatics and comparison of indicated parameters. Areas within yellow and white dashed boxes are magnified in middle and right panels. Scale bars: 200 μm. Each dot indicates a value from 1 mouse, and *n* = 4 mice/group from 2 independent experiments. Data are shown as the mean ± SD; *P* value versus WT by 1-way ANOVA followed by Dunnett’s post hoc test.

We observed that Erg and Fli1 are constitutively expressed in lymphatics in adult tissues and during embryonic and postnatal development (Supplemental Figure 13, A–F). To determine the roles of Erg and Fli1 in lymphatic development, we deleted Erg, Fli1, or Erg/Fli1 in LECs of WT*, Erg*^iΔLEC^*, Fli1*^iΔLEC^, and *Erg/Fli1*^iΔLEC^ embryos by administration of tamoxifen into the pregnant mice at E12.5 and E14.5 and analyzed embryos at E16.5 (Figure 8A). Prominent hemorrhage was noted in the back of the heads of *Erg*^iΔLEC^ and *Erg/Fli1*^iΔLEC^ embryos, and substantial subdermal edema was evident in the back of the head and the back of *Erg/Fli1*^iΔLEC^ embryos (Figure 8B). In contrast, no abnormal phenotypes were observed in WT and *Fli1*^iΔLEC^ embryos. Compared with the WT embryos, *Erg/Fli1*^iΔLEC^ embryos exhibited enlarged, ballooned, and mispatterned lymphatic plexus with 1.9- and 4.0-fold increased lymphatic midline gap and lymphatic length; 42.8%, 60.8%, 45.4%, and 64.3% reduced number of branching points; putative valve-initiating Prox1^hi^ LEC clusters; and Prox1 and Vegfr3 intensities in the lymphatics of the back skin (Figure 8, B–I). Although *Erg*^iΔLEC^ embryos exhibited a 1.8-fold increased lymphatic midline gap and reduced Vegfr3 intensity by 34.9%, the rest of the phenotypes were indifferent in the back skin compared with those of WT mice (Figure 8, B–I). No notable alterations were evident in the back skin of *Fli1*^iΔLEC^ embryos. Thus, Erg alone plays a substantial role in lymphatic development.

To determine the roles of Erg in lymphatic valve development, we deleted Erg or Erg/Fli1 in LECs of WT*, Erg*^iΔLEC^*,* and *Erg/Fli1*^iΔLEC^ embryos by administration of tamoxifen into the pregnant mice at E12.5 and E14.5 and analyzed mesenteric lymphatic valves in embryos at E18.5 (Supplemental Figure 14A). Compared with WT embryos, *Erg*^iΔLEC^ and *Erg/Fli1*^iΔLEC^ mesenteries showed no discernible lymphatic valve structures and marked reductions in the expression of lymphatic valve signatures, including Col IV (60% and 57%), integrin-α9 (63% and 72%), and Vegfr3 (48% and 64%) at the potential valvular areas (Supplemental Figure 14, B and C). Although the expression levels of Foxc2 were comparable, the expression pattern shifted from valve restricted in WT embryos to a broader distribution throughout the lymphangion in *Erg*^iΔLEC^ and *Erg/Fli1*^iΔLEC^ embryos. Thus, Erg alone plays a critical role in lymphatic valve development. Notably, *Erg*^iΔLEC^ embryos exhibited lethality by E20.5 when tamoxifen was administered at E12.5 and E14.5 (Supplemental Figure 14, D and E).

Postnatal mice exhibited similar phenotypes to adult mice following Erg/Fli1 deletion. Tamoxifen administrations from P1 to P3 led to lethality within 1 week exclusively in *Erg/Fli1*^iΔLEC^ pups, while single deletion of either Erg or Fli1 alone did not compromise survival (Supplemental Figure 15, A and B). Functional assessment of mesenteric lymphatic drainage by oral gavage of BODIPY 18 showed a significant reduction (54.9%) in *Erg/Fli1*^iΔLEC^ pups compared with WT pups (Supplemental Figure 15, C–E). Moreover, *Erg/Fli1*^iΔLEC^ pups exhibited deformed mesenteric lymphatic structures, while they remained intact in WT and single-knockout pups (Supplemental Figure 15F).

### Erg and Fli1 are essential for regenerative lymphangiogenesis after injury.

**Figure 9:**
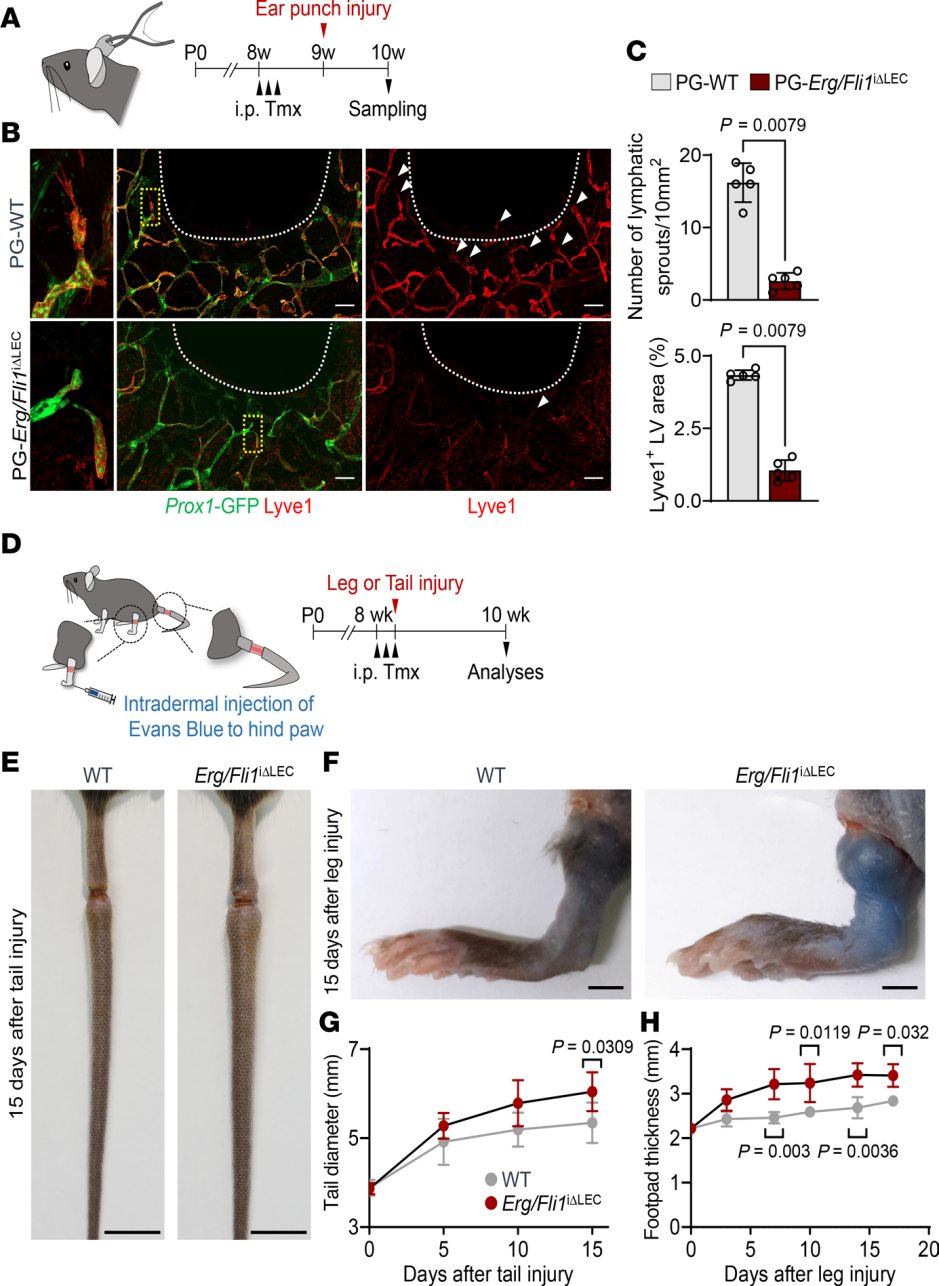
Dual deletion of Erg and Fli1 attenuates regenerative lymphangiogenesis. (**A**) Diagram depicting ear punch injury-induced lymphangiogenesis model, schedules for daily administrations of tamoxifen (Tmx) and ear punch injury, and sampling 2 weeks later. (**B**) Representative images of Prox1^+^/Lyve1^+^ dermal lymphatics after the ear punch injury in PG-WT and PG-*Erg/Fli1*^iΔLEC^ mice. White dashed lines indicate the injury margin, yellow dashed boxes are magnified in right panels, and white arrowheads indicate lymphatic sprouts. Similar findings were observed from *n* = 5 mice/group from 2 independent experiments. Scale bars: 200 μm. (**C**) Comparison of number of lymphatic sprouts and Lyve1^+^ lymphatic density in PG-WT and PG-*Erg/Fli1*^iΔLEC^ mice after the ear punch injury. Each dot indicates a value from 1 mouse, and *n* = 5 mice/group from 2 independent experiments. Data are shown as the mean ± SD; *P* value versus WT by 2-tailed Mann-Whitney *U* test. (**D**) Schematic diagram depicting i.p. administrations of Tmx, generation of tail or leg secondary lymphedema model, and analyses after 2 weeks of injury. (**E**) Representative images of mouse tails after 15 days of injury in WT or *Erg/Fli1*^iΔLEC^ mice. Similar findings were observed from *n* = 5 mice/group from 2 independent experiments. Scale bars: 1 cm. (**F**) Representative images of mouse hind paws after 15 days of injury in WT or *Erg/Fli1*^iΔLEC^ mice. Similar findings were observed from *n* = 3 mice/group from 2 independent experiments. Scale bars: 2 mm. (**G** and **H**) Comparison of tail diameter and footpad thickness in WT and *Erg/Fli1*^iΔLEC^ mice after tail/leg injury models. Each dot indicates a value from 1 mouse, and *n* = 3–5 mice/group from 2 independent experiments. Data are shown as the mean ± SD; *P* value versus WT by 2-way ANOVA followed by Šidák’s post hoc test.

We investigated whether Erg and Fli1 could regulate regenerative lymphangiogenesis in adults. To do this, we performed an ear punch injury 1 week after the deletion of Erg/Fli1 (Figure 9A). Compared with PG-WT mice, PG-*Erg/Fli1*^iΔLEC^ mice exhibited a reduction of 84.0% in lymphatic sprouts and 75.8% in Lyve1^+^ lymphatic area (Figure 9, B and C).

To further assess whether the decreased regenerative lymphangiogenesis could impair lymphatic drainage, we created a secondary lymphedema model in the tail or hind leg (Figure 9D). This was done by disconnecting the lymphatics, which involved excising a circumferential piece of the dermis. Two weeks after the injury, *Erg/Fli1*^iΔLEC^ mice showed a 1.3-fold increase in tail diameter and a 2.0-fold increase in footpad thickness compared with WT mice (Figure 9, E–H). These findings indicate that Erg and Fli1 are crucial for both regenerative lymphangiogenesis and lymphatic recovery.

## Discussion

This study demonstrates that Erg and Fli1 act as essential, cooperative regulators of LEC identity, lymphatic integrity, development, and regenerative capacity. By employing LEC-specific, inducible deletion models in mice, we found that while the loss of either Erg or Fli1 alone was tolerated without overt lymphatic dysfunction, their simultaneous deletion led to relatively rapid-onset, fatal lymphatic failure characterized by chylothorax, chylous ascites, impaired lymphatic drainage, and mortality. These phenotypes underscore a critical redundancy and cooperativity between Erg and Fli1 in maintaining adult lymphatic homeostasis.

Single-cell transcriptomic analysis revealed that codeletion of Erg and Fli1 disrupts the molecular heterogeneity of LECs, resulting in the broad loss of key lymphatic identity markers such as Vegfr3, Lyve1, and Reelin (2). Interestingly, the pan-endothelial junctional molecule PECAM1 showed a marked reduction, implying compromised endothelial junction integrity. This is particularly meaningful in light of recent insights into the dynamic, mechanically sensitive nature of lymphatic junctions (41). Importantly, maintenance of lymphatic valves depends on continuous proliferation and replacement of LECs, which is driven by oscillatory shear stress from lymph flow (12). This mechanical stimulus is essential for activating mTORC1 signaling necessary for valve integrity (17). In *Erg/Fli1*^iΔLEC^ mice, lymphatic coagulation and impaired lymphatic drainage decrease shear stress on the valves. Accordingly, in our single-cell analysis, valve-forming LECs lost the selective expression of valve-specific regulators (Foxc2, Gata2, Gja4, and Nfatc1) (10), suggesting a breakdown in the spatial and transcriptional control required for proper valve differentiation and integrity. This was accompanied by ectopic expression of Foxc2 throughout the lymphangion and morphological valve disruption, highlighting the role of Erg/Fli1 in restricting valve differentiation cues to appropriate anatomical domains.

Mechanistically, we identified that Erg directly binds to conserved gene regulatory regions of *Ccl21* and is required for its expression in LECs, linking Erg/Fli1 function to immune cell trafficking and homeostatic immune surveillance. The near-complete loss of Ccl21 in Erg/Fli1-deficient lymphatics resulted in impaired DC migration, further supporting the importance of these transcription factors in lymphatic-immune crosstalk.

Our findings show that DC trafficking to the draining lymph node was significantly but modestly reduced in *Erg/Fli1*^iΔLEC^ mice, which have almost no Ccl21 in the lymphatics. Although Ccl21/Ccr7 signaling is known to promote DC migration toward lymphatics and lymph nodes (32–35), multiple signaling pathways are involved in DC trafficking (37, 42). Ccl19 produced from stromal cells in the secondary lymphoid organs binds to Ccr7 on DCs to guide their migration to lymph nodes in the adaptive immune response (35, 43). In this process, the Ccl19 action is greater than that of Ccl21 (37). Under inflammatory conditions, both the Ccl21/Ccr7 and Ccl21/Ccr7-independent signaling pathways promote DC migration (42). Moreover, the elevation of chemokines (Cxcl1, Ccl2, and Ccl7) in the LECs may reduce DC trafficking and migration to lymph nodes in *Erg/Fli1*^iΔLEC^ mice. Thus, these potential mechanisms could lead to the modest reduction of DC trafficking to the draining lymph node in *Erg/Fli1*^iΔLEC^ mice. In addition to identity and immune function, Erg/Fli1 codeletion induced a pathological shift toward proinflammatory and prothrombotic states, as evidenced by the upregulation of chemokines (*Cxcl1* and *Ccl7*) and coagulation-related genes (*Serpine1, F2r,* and *Pf4*) (40, 44). Elevated Serpine1 protein levels in lymph and chyle coagulation in affected mice directly implicate Erg/Fli1 in suppressing thrombogenic pathways within the lymphatic system.

Notably, Erg has also been identified as a crucial regulator of cardiac valve development (34), implying its broader impact in specialized valvular programs across vascular systems. Developmentally, embryos lacking both Erg and Fli1 in LECs exhibited severe lymphatic plexus mispatterning, reduced branching, and a loss of valve-initiating Prox1^hi^ clusters. In comparison, Erg or Fli1 deletion alone had a substantial or minimal impact. This suggests that while Erg is required for adequate embryonic lymphatic and lymphatic valve development, Fli1 may compensate for Erg deficiency during embryonic development. Consistently, adult mice lacking both factors displayed severely impaired regenerative lymphangiogenesis and exacerbated secondary lymphedema following injury, highlighting their necessity for lymphatic repair and adaptation.

Our findings align with and extend previous work in BECs, where Erg and Fli1 have been shown to regulate vascular stability, angiogenesis, and homeostasis (20). The present study provides direct evidence that these factors are equally critical in lymphatic endothelium, with unique roles in maintaining vessel identity, valve integrity, and the immune-vascular interface. The disruption of valve identity and function in ERG/FLI1-deficient lymphatics phenocopies aspects of lymphedema-distichiasis syndrome caused by FOXC2 mutations (13). Moreover, a recent human genome-wide association study for rare disease revealed genetic variants in ERG that strongly correlate with primary lymphedema, preventing transcriptional function of ERG by their mislocalization into the cytosol (45). This evidence suggests that ERG/FLI1 dysfunction contributes to human lymphatic disorders.

Despite these advances, open questions remain. The precise molecular interactions between Erg/Fli1 and established lymphatic regulators such as Prox1 and Vegf-c/Vegfr3 signaling, and how these regulate lymphatic-specific transcriptional profiles and genome-wide accessibility, require further investigation. Additionally, the mechanisms by which Erg/Fli1 suppress prothrombotic gene expression in LECs and their potential involvement in human coagulation disorders represent important avenues for future research.

In summary, this study identifies Erg and Fli1 as central, cooperative regulators of lymphatic vessel integrity, valve specialization, and regenerative lymphangiogenesis. Their loss leads to fatal lymphatic failure, driven by loss of identity, valve dysfunction, and a shift toward proinflammatory and prothrombotic states. These findings not only advance our understanding of lymphatic biology but also highlight Erg/Fli1 as potential therapeutic targets in lymphatic vascular diseases and related coagulopathies.

## Methods

### Sex as a biological variable.

Mice of both sexes were used for all experiments. As sex-specific differences were not observed, data from both sexes were combined for analysis. Hence, we did not consider sex as a biological variable.

### Animals.

*Prox1*-CreER^T2^ (28) mice were obtained from T. Mäkinen (Wihuri Research Institute, Helsinki, Finland), and *Prox1*-GFP mice (27) mice were obtained from Y.K. Hong (Keck School of Medicine of the University of Southern California, Los Angeles, California). *Erg*^fl/fl^ mice and *Fli1*^fl/fl^ mice were generated by in vitro fertilization of embryonic stem cells purchased from the Knockout Mouse Project. All mouse strains were bred and maintained under specific pathogen-free conditions at Korea Advanced Institute of Science and Technology, and they were fed a standard chow diet (PMI LabDiet) with water. Experiments were performed during the light period. The mice were anesthetized by i.p. injection of ketamine (10 mg/kg) and xylazine (1 mg/kg) before procedures. Supplemental anesthesia was given as necessary during procedures. Body temperature was maintained at 36.5°C–37.5°C during surgical and imaging procedures.

### Sample preparations for histological analyses and immunofluorescence staining.

Mice were anesthetized and perfused with ice-cold PBS followed by 2% formalin (HT501128, Sigma-Aldrich) through the left ventricle after puncturing the right auricle. Dorsal skin, mesentery, ear skin, inguinal and parotid lymph nodes, diaphragm, trachea, and mitral valves were harvested and postfixed in 2% formalin at 4°C overnight. The inguinal and parotid lymph nodes were sectioned into 20-μm-thick slices using a cryotome (CM3050S, Leica), while other tissues were whole mounted. Samples were washed with PBS and blocked with 5% serum (Jackson ImmunoResearch) in 0.2% Triton X-100 in PBS for 1 h at room temperature (RT).

The samples were then incubated with the following primary antibodies diluted in the blocking solution at 4°C overnight: anti-ERG (rabbit monoclonal, ab92513, Abcam); anti-Fli1 (rabbit monoclonal, ab133485, Abcam); anti-CCL21 (goat polyclonal, AF457, R&D Systems); anti-CD31 (hamster monoclonal, MAB1398Z, Merck); anti-VEGFR3 (goat polyclonal, AF743, R&D Systems); anti-LYVE1 (rabbit polyclonal, 11-034, Angiobio); anti-Reelin (goat polyclonal, AF3820, R&D Systems); anti-FOXC2 (sheep polyclonal, AF6989, R&D Systems); anti–Integrin-α9 (goat polyclonal, AF3827, R&D Systems); and anti-NFATC1 (goat polyclonal, AF5640, R&D Systems). After several washes with PBS, the samples were incubated with Alexa Fluor 488–, 594–, or 647–conjugated secondary antibodies (Jackson ImmunoResearch) diluted 1:1,000 in the blocking solution at RT for 4 h: anti-rabbit (code numbers 711-545-152, 711-585-152, and 711-605-152), anti-goat (code numbers 705-545-147, 705-585-147, and 705-605-147), anti-sheep (code numbers 713-545-147, 713-585-147, and 713-605-147), and anti-hamster (code numbers 127-545-160, 127-585-160, and 127-605-160). After several washes with PBS, samples were mounted with Vectashield (Vector Laboratories).

### Imaging and morphometric analyses.

Immunofluorescent images were acquired using an LSM880 confocal microscope (Carl Zeiss). ZEN 2.3 software (Carl Zeiss) was used to acquire and process images. Confocal images of stained samples were processed with maximum intensity projections of single-plane *Z*-stack images through the 10 to 15 μm thickness of tissues, which were all taken at a resolution of 1,024 × 1,024 pixels with the LD C-Apochromat ×40/1.1 NA water immersion Corr M27 (LSM 880) with multichannel scanning in the frame. 3D reconstruction images were created from *Z*-stack confocal images using the 3D tab in ZEN 2.3 software. Images were obtained from 3 different portions of each sample, and Erg- or Fli1-positive LECs in each tissue were counted manually using ZEN 2.3 software. Fluorescence intensities of Col IV, integrin-α9, Vegfr3, and Foxc2 in the potential lymphatic valve area (70 μm ×70 μm) were quantified from confocal images with ZEN 2.3 software.

### Immunoblotting assay.

To deplete ERG and/or FLI1 in human LECs, control siRNA (D-001810-10-05, Dharmacon), ERG siRNA (L-003886-00-0005, Dharmacon), or Fli1 siRNA (L-003892-00-0005, Dharmacon) was transfected with Lipofectamine RNAiMAX Transfection Reagent (13778100, Thermo Fisher Scientific). At 48 h after transfection, human LECs in RIPA buffer (CBR002, LPS Solution) containing protease inhibitor and phosphatase inhibitor (5872, Cell Signaling Technology) were scraped with a cold plastic cell scraper and agitated for 30 min at 4°C until tissues were lysed, followed by centrifugation at 13,000 rpm for 10 min at 4°C. The total protein isolated from each sample was measured and normalized with a Pierce BCA Protein Assay Kit (23225, Thermo Fisher Scientific). An equal amount of protein from tissue lysate was loaded into each well of a 7.5%–12% SDS-polyacrylamide gel after denaturation with SDS loading buffer for 5 min at 95°C. After electrophoresis, proteins were transferred to a PVDF membrane, incubated with 2% BSA blocking buffer for 1 h at RT, and blotted with the following antibodies overnight: rabbit anti-ERG (ab92513); rabbit anti-Fli1 (ab133485) (Abcam); mouse anti-VEGFR3/Flt4 (MAB3491); sheep anti-FoxC2 (AF5044); goat anti-VEGFR2/Flk-1 (AF357); sheep anti-CD31 (AF806); goat anti-GATA2 (AF2046) (R&D Systems); mouse anti-NFATC1 (MA3-024, Invitrogen); and rabbit anti-GAPDH (2118, Cell Signaling Technology). After several washes with TBST solution (CBT007, LPS Solution), the membrane was incubated with anti-rabbit (7074, Cell Signaling Technology), anti-mouse (7076, Cell Signaling Technology), anti-sheep (HAF016, R&D Systems), or anti-goat (HAF109, R&D Systems) secondary peroxidase–conjugated antibody for 1 h at RT. Target proteins were detected using ECL western blot detection solution (WBKLS0500, Millipore). The same amount of protein loading in each lane was verified by immunoblotting with GAPDH. Images were obtained using the Amersham Imager 600 (GE Healthcare Life Sciences), and protein density was measured using Image Studio Lite (LI-COR).

### ChIP-qPCR.

Primary cultured human LECs were fixed with 0.5% formalin for 5 min at 37°C and then quenched with 0.1 M glycine for 5 min at RT. Samples were lysed with lysis buffer containing 1% SDS, 10 mM EDTA, 50 mM Tris (pH 7.9), protease inhibitor, and phosphatase inhibitor. The fixed DNA in the cell lysates was fragmented by sonication using a focused ultrasonicator (Covaris). The cell lysates were centrifuged, and the resulting supernatants were further diluted with ChIP dilution buffer containing 0.1% Triton X-100, 2 mM EDTA, 20 mM Tris (pH 7.9), 150 mM NaCl, protease inhibitor, and phosphatase inhibitor. As whole-cell lysate input, 5% volume was saved. Diluted samples were then incubated with ERG antibody (mouse anti-ERG1/2/3, sc-271048, Santa Cruz Biotechnology) and protein A/G Dynabeads (Thermo Fisher Scientific) at 4°C overnight. The beads were isolated with DynaMag-2 (Thermo Fisher Scientific) and washed with a series of ChIP wash buffers: low-salt wash buffer, high-salt wash buffer, LiCl wash buffer, and TE buffer. Samples were eluted in ChIP elution buffer containing 1% SDS and 0.1 M NaHCO_3_ for 15 min 2 times at 65°C. Beads were removed, and the eluted samples and 5% whole-cell lysate input were reverse-cross-linked at 65°C overnight. After normalizing pH, samples were incubated with RNase (3 μg/mL) for 2 h at 37°C and then for 2 h with proteinase K (20 mg/mL) and glycogen (20 mg/mL) at 55°C. DNA was eluted using a standard procedure. Input and ChIP-DNA for *CCL21* were analyzed by qPCR using primers listed in Supplemental Table 1.

### Micro-CT.

Micro-CT was performed with a Quantum GX micro-CT imaging system (PerkinElmer) to assess the pulmonary edema in the mice. The mice were anesthetized with 1.5% isoflurane, and respiration was monitored during the scan. Micro-CT acquisition parameters were set to 90 kV, 160 mA, and 12 ms exposure per projection. 512 projections were acquired with a 0.725° increment for a total rotation angle of 370°. For imaging, horizontal sections above the liver contour were selected.

### Thoracic duct lymphangiography.

After the mice were anesthetized, 20 μL of Evans blue dye (1% w/v) was injected intradermally into the right hind paws. The thoracic cavity was opened after 15 min of injection, and 50 mL of 4% paraformaldehyde (PFA) in PBS at 4°C was perfused through the left ventricle serially. The aorta and surrounding fat tissue were removed carefully, just above the diaphragm to the aortic arch. For measurement of the width of the thoracic duct, the whole thoracic cavity was fixed in 4% PFA in PBS at 4°C for 6 h. After dissecting the thoracic duct, gross images were acquired using an AxioZoom V16 stereo zoom microscope (Carl Zeiss).

### Inguinal lymph node drainage assay.

After the mice were anesthetized, using microsurgery curved scissors, a midline incision was made from the pubis to the xiphoid process, and the skin was dissociated from the abdominal muscle to unveil the inguinal lymph node. 10 μL of Evans blue dye (1% w/v) was directly injected into the middle of the lymph node using a syringe with a 31-gauge needle. After 30 s, the gross images of Evans blue dye drainage were acquired using the AxioZoom V16 microscope.

### Lipid absorption and mesentery lymphangiography.

Neonatal (P5) mice were fasted for 5 h and gavaged with 10 μL of BODIPY 493/503 (Invitrogen) (250 μg/mL) diluted in olive oil on a warming pad. 30 min after gavage, mice were anesthetized, and mesenteries were harvested. Mesenteries were pinned on a silicon plate and washed with ice-cold PBS. After washing, the fluorescent images of mesenteries were acquired using the AxioZoom V16 microscope.

### ELISA.

After the mice were anesthetized, the abdominal cavity was opened to expose the superior mesenteric arteries and surrounding mesenteric lymph ducts. The lymphatics were cannulated with a 32-gauge needle, and lymph was collected using a 10 μL microsyringe (80330, Hamilton). ELISA was used to measure the concentration of SERPINE1 in collected lymph with a mouse SERPINE1-specific ELISA kit (ab197752, Abcam).

### Culture of BMDCs.

Following a previous study (37), bone marrow cells were isolated from femur and tibia of C57BL/6J mice and cultured in the DC medium containing RPMI, 10% FBS, 15 nM HEPES, 1 mM sodium pyruvate, 100 U/mL penicillin, 100 μg/mL streptomycin, 2 nM l-glutamine (GIBCO), 50 μg β-mercaptoethanol (Sigma-Aldrich), and 40 ng/mL GM-CSF (R&D Systems). On day 9, cells were stimulated with 0.2 μg/mL lipopolysaccharide (Sigma-Aldrich) in the DC medium for 24 h. The purity of differentiated DC was assessed by flow cytometry using DAPI (422801, BioLegend), anti-mouse CD45-BV711 (103147, BioLegend), anti-mouse CD11c-PE (117308, BioLegend), and anti-mouse I-A/I-E-APC (107614, BioLegend). Purified DCs were labeled with 2.5 μM CellTracker Red CMTPX (C34522, Invitrogen) in DC medium for 30 min at 37°C. After several washes with PBS, DCs were resuspended in PBS and intradermally injected into the right ear skin (75,000 cells/mouse), and the ipsilateral parotid lymph node was sampled at 4 h after the injection.

### Ear punch lymphangiogenesis model.

2 mg of tamoxifen was i.p. injected for 3 consecutive days in 8-week-old mice. Just after 3 days of tamoxifen injection, a 1.5 mm hole was made in the center of both ears of mice using a metal ear punch (Harvard Apparatus). Two weeks after punching, mice were anesthetized with an i.p. injection of a combination of anesthetics (80 mg/kg of ketamine and 12 mg/kg of xylazine). Ear skin was harvested (with hair removal), fixed in 4% PFA in PBS at 4°C for 2 h, and washed 3 times in PBS.

### Tail and hind limb lymphedema mouse models.

For a tail lymphedema model (46), the mice were anesthetized with 1%–3% isoflurane inhalant, and a circumferential 2-mm-wide piece of the dermis was excised located 1 cm distal from the tail base, leaving all the underlying vasculature intact under a dissecting microscope. Then, the mice were sanitized with povidone-iodine, and an antibiotic cream was applied on the excision site for 7 consecutive days. For the tail diameter, the thickest diameter from the distal part of the excision site was measured. For a hind limb lymphedema model (47), mice were anesthetized with 1%–3% isoflurane inhalant, and the circumferential skin of the inguinal crease was excised. 10 μL of Evans blue dye (1% w/v) was injected intradermally into the plantar surface of hind paws to visualize the popliteal node and lymphatic drainage. The popliteal node and the following lymphatic vessels were dissected and removed, leaving the adjacent vein carefully. Then, a skin suture was made with a 2–3 mm gap between each skin margin side. Lymphedema was assessed and evaluated by measuring the paw thickness using an electronic caliper.

### Droplet-based scRNA-seq of mesenteric lymphatics.

After anesthesia, WT mice (8–12 weeks) and *ERG/Fli1*^iΔLEC^ mice (8–12 weeks) were perfused with ice-cold PBS. Intestines with intact mesenteries were removed by cutting the stomach and rectum and kept in ice-cold DMEM/F12 medium (Gibco). The mesenteries were collected by carefully cutting along the intestine. Mesenteries were mechanically cut into small pieces by scalpel and incubated in dissociation buffer containing 2 mg/mL of collagenase II (Worthington), 1 mg/mL of dispase (Gibco), and 0.2 mg/mL DNase I (Gibco) at 37°C for 30 min with gentle pipetting up and down every 10 min. Digested tissues were immediately centrifuged at 300*g*, and fat-containing supernatants were removed. Then, an equal volume of 10% FBS/PBS was added to resuspend the pellet and filtered through a 70 μm strainer. The cells were further centrifuged for 10 min with 300*g* and resuspended in PBS containing 2% FBS. The resuspended cells were incubated with a cocktail of anti-CD45, anti-EPCAM, and anti-PDGFRα antibody-conjugated microbeads on ice for 15 min to discard immune, epithelial, and stromal cells using magnetic columns. Then, dead cells were removed by FACS (FACSAria Fusion, BD Biosciences) after staining with DAPI (564907, Sigma-Aldrich). Subsequently, GFP^+^ cells were sorted and resuspended in 2% FBS/PBS. Following the manufacturer’s protocol, cells were processed using the Chromium Next GEM Single Cell 3p RNA library kit v3.1 (10x Genomics) to make single-cell libraries. Briefly, single-cell suspensions were loaded and separated into single cells as a gel bead in emulsion. Then, for individual cells, transcripts were reverse transcribed with unique barcodes and amplified to generate cDNA libraries. SPRI beads (Beckman Coulter) were used to filter libraries in appropriate sizes. Filtered cDNA libraries were fragmented and ligated with adaptors and amplified with index primers unique to samples. Amplified products were double-sided size selected by SPRI beads, and their qualities were checked using the Agilent Bioanalyzer High Sensitivity Chip. The libraries that passed quality controls were sequenced by the NovaSeq platform (Illumina).

### Processing of scRNA data.

Sequenced libraries were demultiplexed to produce Fastq files and aligned to a customized mouse reference genome (mm10) that included eGFP sequence, using the cellranger counts function from Cell Ranger (version 3.0.2). The resulting outputs were then processed into a count matrix using the Read10X function in the R package Seurat (version 3.1.1) (48). For cell-level quality control, cells with fewer than 1,200 detected genes, with over 8,000 genes, or with over 10% of reads mapping to mitochondrial genes were excluded, as these were considered low-quality cells, doublets, or dead cells, respectively. For gene-level filtering, genes expressed in fewer than 3 cells were removed from the raw expression matrix. Following this quality filtering, expression matrices were normalized by dividing each gene’s unique molecular identifier (UMI) count by the total UMI count per cell, multiplying by a scale factor of 10,000, and applying a log_2_ transformation to approximate log counts per million.

### Clustering analysis.

Clustering and subsequent analyses were performed using the Seurat R package (version 3.1.1). The 2,000 most highly variable genes for each dataset were selected by the FindVariableFeatures function with the option, selection.method = ‘vst’. Principal component analysis was conducted for initial dimensionality reduction, and the top 15 principal components were retained for downstream analysis, including 2D visualization using UMAP and construction of shared nearest neighbor graph for neighbor detection. Clustering was performed by the Louvain algorithm. Following the initial clustering, cells expressing *Cdh5*, *Pecam1*, and *Prox1* were defined as LECs and retained for subclustering analysis. Additionally, a small number of cells positive for *Pdgfrb* and *Acta2* were considered doublets and excluded. LECs from each dataset were downsampled to 3,000 cells to equalize cell numbers across datasets. The remaining LECs were reprocessed through dimensionality reduction and clustering to identify LEC subtypes. To determine DEGs across clusters or datasets, the FindMarkers function was applied with the following parameters: test.use = ‘MAST’, min.pct = 0.3, and logfc.threshold = 0.5.

### Comparison with bulk RNA-seq data.

Bulk RNA-seq data of BECs following Erg/Fli1 deletion were obtained from a previous study under accession code GSE210119 (20). DEGs identified in *ERG/Fli1*^iΔLEC^ mice in this study were classified as upregulated or downregulated and compared with corresponding DEG sets from BECs to identify overlapping and cell type–specific genes regulated by Erg/Fli1.

### RT-PCR.

Total RNA was extracted using TRIzol isolation reagent (Invitrogen) using the manufacturer’s instructions. RNA concentration was determined using a NanoDrop spectrophotometer (Thermo Fisher Scientific). Using murine leukemia virus reverse transcriptase and oligo (dT)16 primer, 2 μg of cell RNAs was reverse transcribed. The resulting cDNAs from samples were assayed in duplicate. RT-PCR was conducted using 2× SYBR green PCR master mix on a real-time PCR system (Applied Biosystems). Gene expression data were normalized to the housekeeping gene *GAPDH* and analyzed using the ΔΔCt method. Primer sets were designed using the PrimerQuest tool (Integrated DNA Technologies): *CCL21* (5′-CTCAAGTACAGCCAAAGGAAGA-3′, 5′-CAAGAACAGGATAGCTGGGATG-3′); *GAPDH* (5′-GGTGTGAACCATGAGAAGTATGA-3′, 5′-GAGTCCTTCCACGATACCAAAG-3′).

### Echocardiography assessment.

Cardiac function was evaluated via transthoracic echocardiography using the Prospect 3.0 High-Resolution Imaging System (version 3.2.1, S-Sharp Corporation). Mice were anesthetized with 1%–2% isoflurane, placed in a supine position, and secured to a temperature-controlled platform with adhesive tape. ECG signals were continuously monitored via 3 limb electrodes (left forelimb and both hind limbs) and recorded using the Harvard Apparatus Monitoring System (version 3.3.2). Long-axis views of the heart were acquired at the midventricular level, standardized by visualization of the aortic valve. M-mode images were obtained to measure left ventricular internal diameters at end-diastole and end-systole and to calculate ejection fraction, fractional shortening, stroke volume, and left ventricular mass (49, 50).

### Assessment of vascular permeability in the mesentery.

To evaluate capillary permeability, the mice were anesthetized, and a small abdominal incision was made to expose the mesenteric vein and surrounding capillaries. A portion of the mesentery was gently spread over a glass slide and kept moist with sterile saline. Evans blue dye (1% in PBS, 50 μL) (E2129, Sigma-Aldrich) was administered via the tail vein, and fluorescence images of mesenteric blood vessels were acquired at the indicated time points using an AxioZoom V16 microscope. Fluorescence intensity was quantified with ZEN 2.3 software to assess vascular permeability.

### Statistics.

Sample sizes were chosen on the basis of standard power calculations (with α = 0.05 and power of 0.8). No statistical methods were used to determine sample size. Experiments were randomized, and investigators were blinded to allocation during experiments and outcome assessment. Depending on the data distribution, parametric or nonparametric statistical tests were performed. The statistical significance of differences was determined by 1-way ANOVA followed by Dunnett’s post hoc test, 2-way ANOVA followed by Šidák’s post hoc test, 2-tailed Welch’s *t* test, or 2-tailed Mann-Whitney *U* test. Statistical analyses were performed using Prism 10 (GraphPad Software, version 10.2.3). All data are shown as the mean ± SD. Statistical significance was set at *P* < 0.05.

### Study approval.

All animal care and experimental procedures were approved by the Institutional Animal Care and Use Committees of the Korea Advanced Institute of Science and Technology (KA2025-127-v1) and Institute for Basic Science (IBS-2025-050).

### Data availability.

The scRNA-seq data of this study are available in the NCBI’s Gene Expression Omnibus under accession code GSE296184. The R codes for the analysis in the current study are available from the corresponding authors upon reasonable request. Values for all data points in graphs are compiled in the Supporting Data Values file.

## Author contributions

MJY, HJL, SK, and GYK conceived and designed the study. MJY, SPH, SK, and HJL performed most of the experiments and generated the figures with contributions from CJ, CMY, SHY, JJ, and LGG. MJY, HJL, and GYK wrote the manuscript with contributions from SPH and SK under the supervision of GYK. MJY, HJL, SPH, SK, and GYK analyzed and interpreted the data and generated the figures. MJY performed scRNA-seq and analyzed scRNA-seq datasets. MJY, HJL, SPH, SK, HJ, and JHY discussed the experiments and results under the supervision of GYK. GYK supervised and directed the project.

## Funding support

- Institute of Basic Science.
- Ministry of Science and ICT, South Korea (IBS-R025-D1-2015), to GYK.

## Supplementary Material

Supplemental data

Unedited blot and gel images

Supporting data values

## Version 1 12/29/2025

In-Press Preview

## Version 2 03/02/2026

Electronic publication
