# Supplementary material for: Cooperative ETS transcription factors are required for lymphatic endothelial cell integrity and resilience
Source: J Clin Invest. 2025 Dec 23;136(5):e196119. doi: 10.1172/JCI196119 (PMC13067931; doi:10.1172/JCI196119)
Supplement: Supplemental data [file jci-136-196119-s010.pdf]

Supplemental Data

Supplemental Figures and Legends (1-15)

Supplemental Table 1

Cooperative ETS transcription factors are required for lymphatic endothelial cell integrity and resilience

Myung Jin Yang, Seok Kang, Seon Pyo Hong, Hokyung Jin, Jin-Hui Yoon, Cheol Hwa Jin, Chae Min Yuk, Lydia Getachew Gebeyehu, Junho Jung, Sung-Hwan Yoon, Hyuek Jong Lee and Gou Young Koh

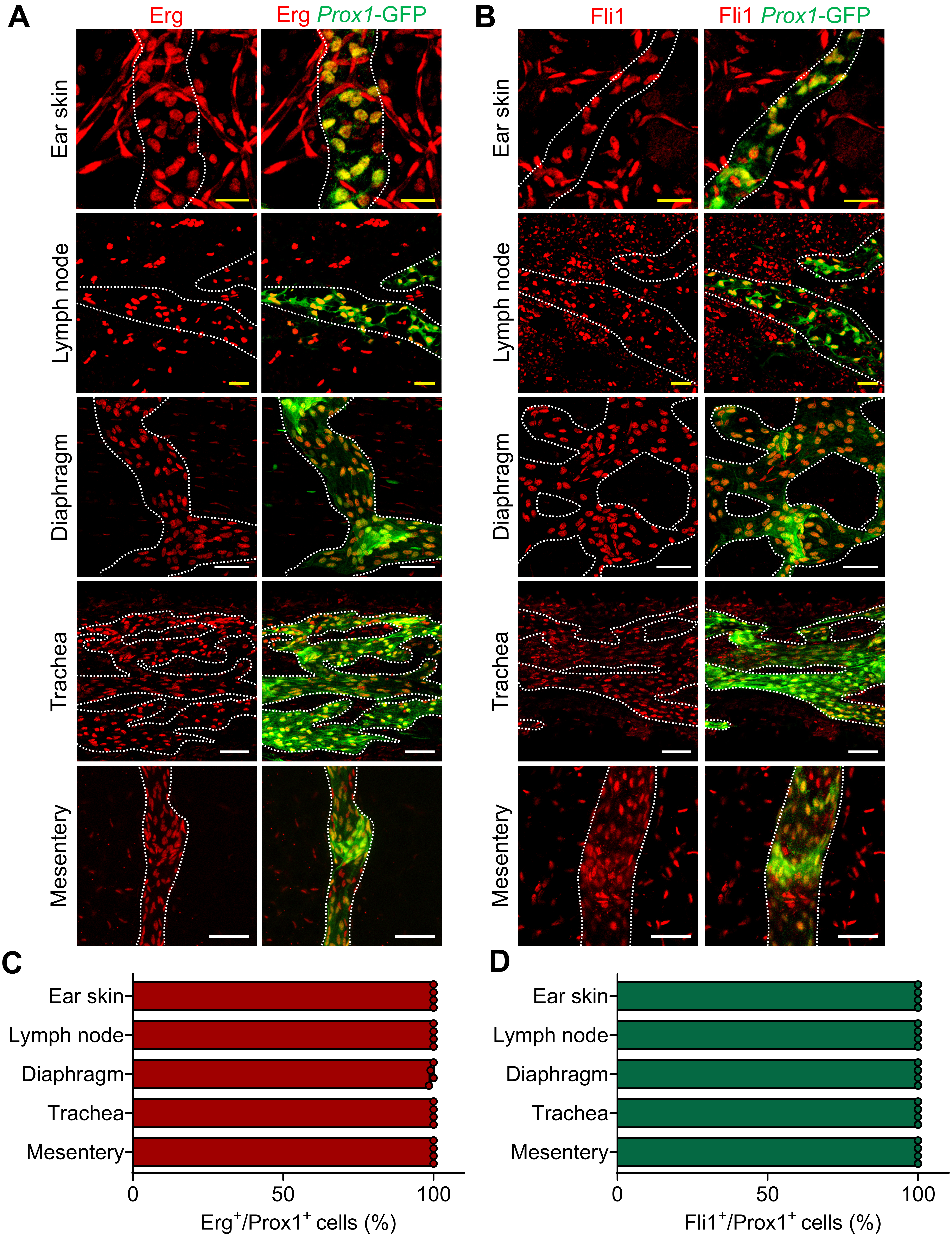

Supplemental Figure 1. Erg and Fli1 are constitutively present in the nuclei of lymphatic endothelial cells in various organ lymphatics of adult mice

A-D, Immunofluorescence images and comparisons for Erg and Fli1 in the nuclei of blood and lymphatic (marked with white dashed lines based on *Prox1*-GFP$^+$) endothelial cells in the ear skin, lymph node, diaphragm, trachea, and mesentery of 8-week-old mice. Yellow scale bars, 20 μm. White scale bars, 50 μm. Each dot indicates a value from one mouse and n = 4 mice/group from two independent experiments. Horizontal bars indicate mean ± SD.

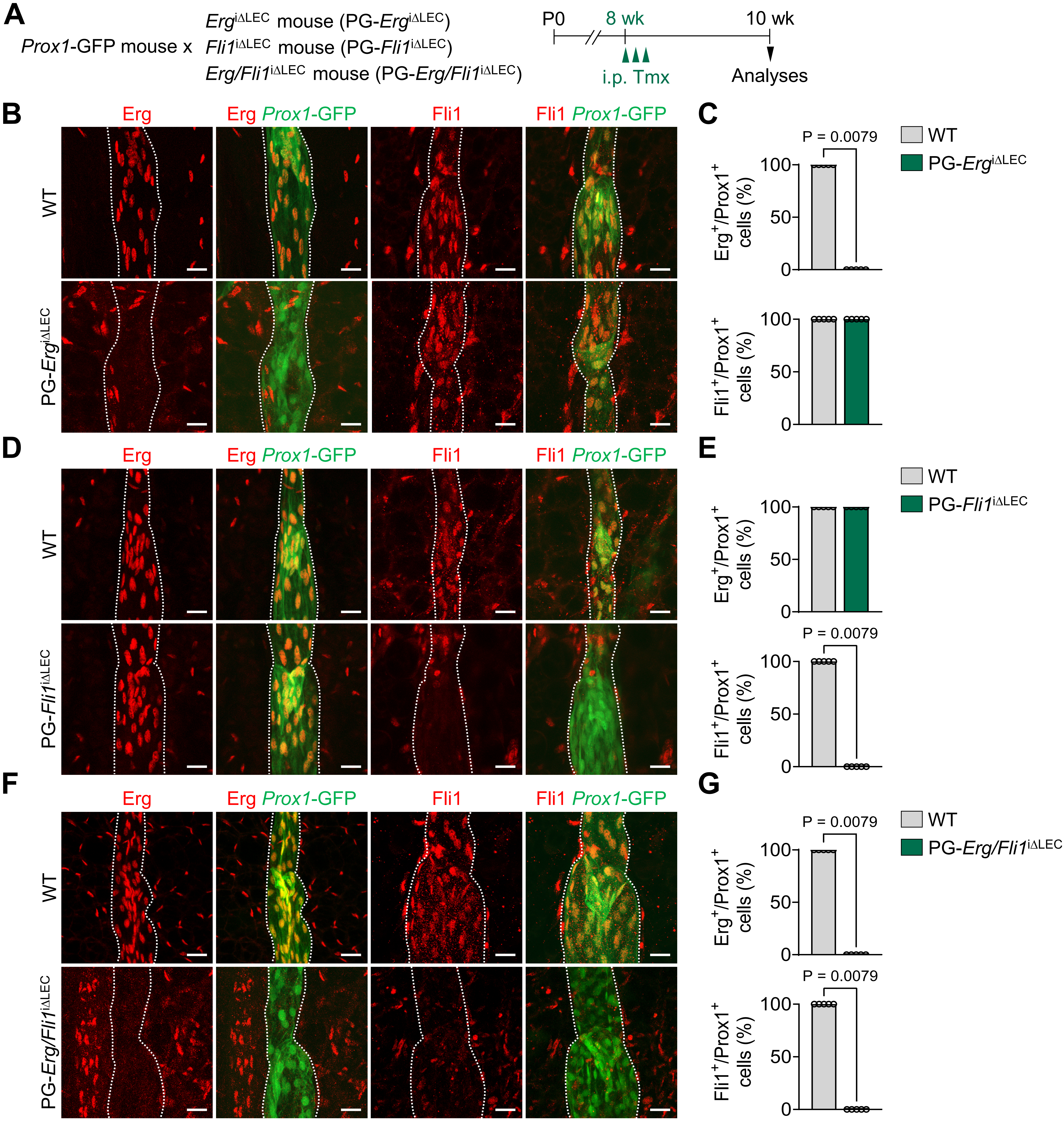

Supplemental Figure 2. Deletion efficiency of Erg and Fli1 in each lymphatic-specific conditional knock-out mice

A, Diagram showing schedule of intraperitoneal Tmx administrations for three consecutive days and analyses at 2 weeks after the first Tmx injection in each indicated mouse line. **B-G,** Representative images and percentages of Erg^+ or $Fli1^+$ LEC in mesenteric lymphatics (white dashed lines) of the indicated mouse line. Scale bars, 20 μm. Each dot indicates a value from one mouse and n = 5 mice/group from two independent experiments. *P* values versus WT by two-tailed Mann-Whitney *U* test.

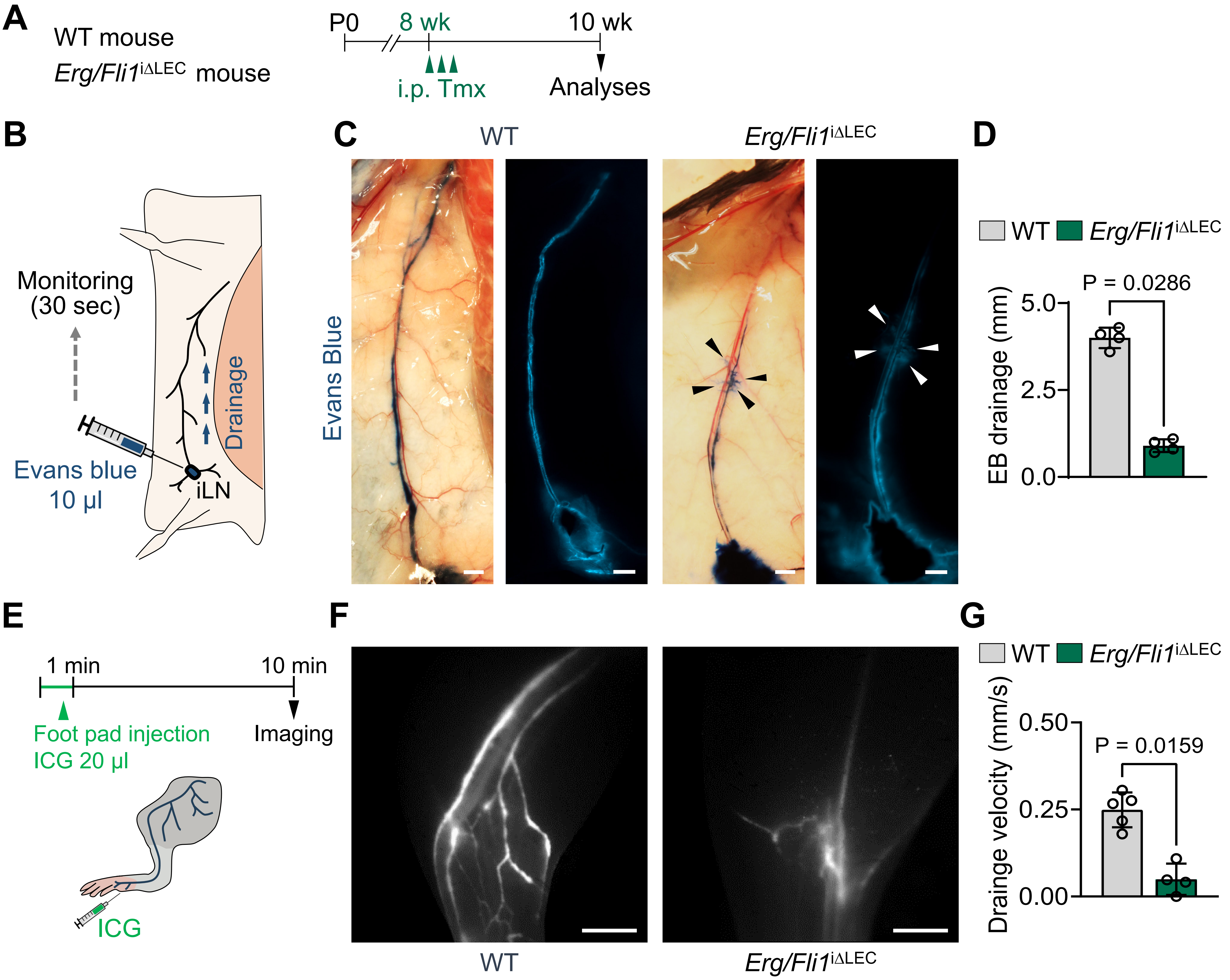

Supplemental Figure 3. Erg and Fli1 double knockout impairs lymphatic drainage function

A-F, (**A**) Diagram showing i.p. administrations of Tmx for three consecutive days to WT and *Erg/Fli1*$^{i\Delta LEC}$ mice for lymphatic drainage assays. (**B**) Diagram depicting lymphatic drainage assay. Ten μl of Evans blue was injected to inguinal lymph node and its drainage through the skin lymphatics was assessed at 30 seconds later. (**C**,**D**) Representative images of skin lymphatic drainage of Evans Blue in WT and *Erg/Fli1*$^{i\Delta LEC}$ mice. Scale bars, 2 mm. Comparison of distances of Evans blue drainage from the inguinal lymph node. Each dot indicates a value from one mouse and n = 4 mice/group from two independent experiments. Bars indicate mean ± SD and *P* values versus WT by two-tailed Mann-Whitney *U* test. (**E**) Diagram depicting thoracic duct lymphangiography. 20 μl of indocyanine green (ICG) was injected for 1 min in right hind footpads, and lymphatics in the thigh were imaged after 10 min. (**F**,**G**) Representative images of lymphangiography in WT and *Erg/Fli1*$^{i\Delta LEC}$ mice. Scale bars, 2 mm. Comparison of lymphatic drainage velocities. Each dot indicates a value from one mouse and n = 4-5 mice/group from two independent experiments. Bars indicate mean ± SD and *P* values versus WT by two-tailed Mann-Whitney *U* test.

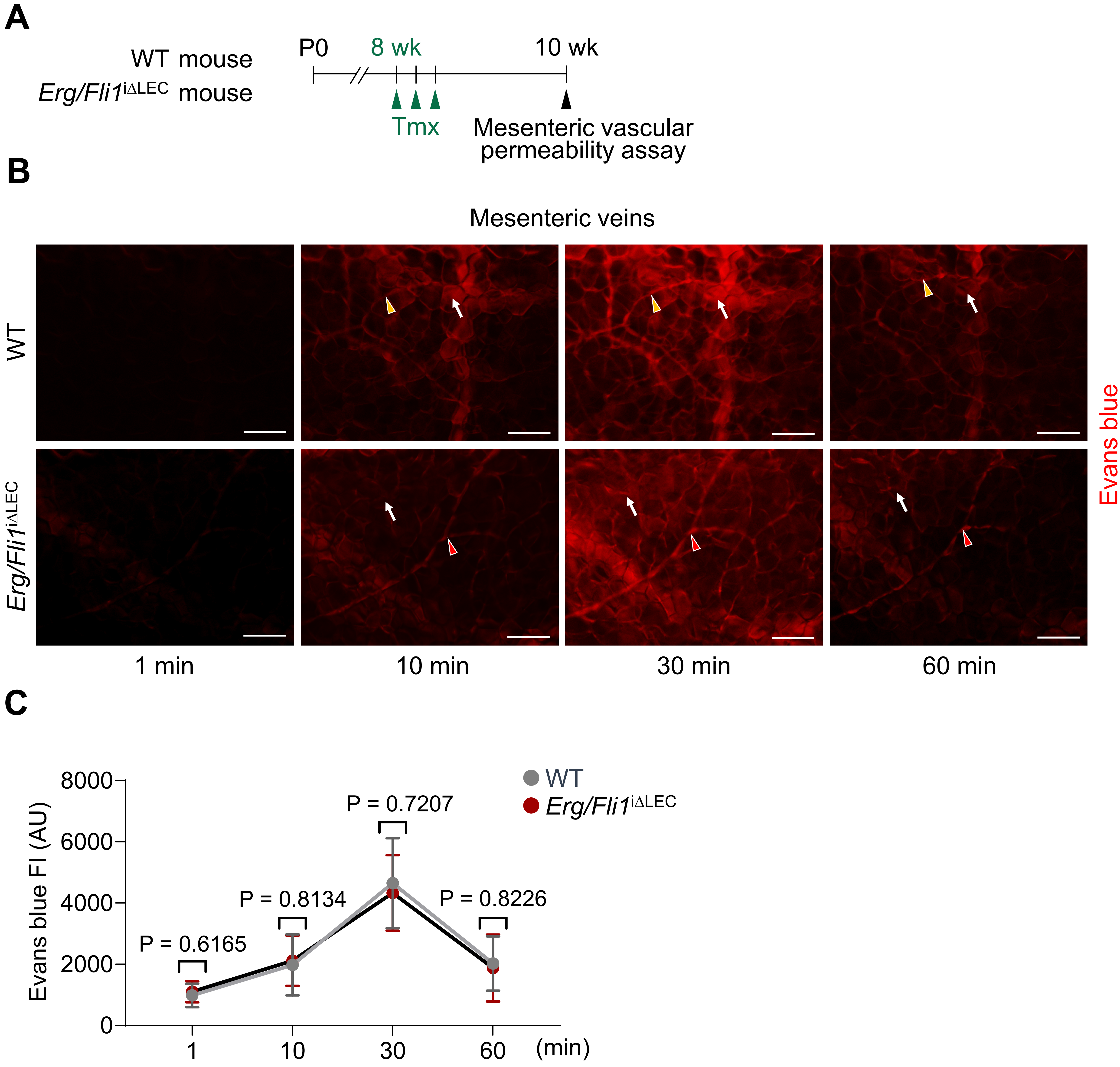

Supplemental Figure 4. Erg and Fli1 depletion in LEC does not compromise blood vessel permeability

A, Diagram of Tmx administration for three consecutive days and intravenous injection of Evans blue (1%, 50 μl) via the tail vein for assessing blood vessel leakage in the mesentery of WT and *Erg/Fli1*$^{i\Delta LEC}$ mice at 2 weeks post-injection of initial Tmx.

B, Images of Evans blue fluorescence (red signal) in the mesenteric vasculatures (yellow and red arrowheads) beneath adipocytes (white arrows) of WT and *Erg/Fli1*$^{i\Delta LEC}$ mice at the indicated time points after Evans blue injection. Scale bars, 100 μm.

C, Comparison of Evans blue fluorescence intensity (FI) in the mesenteric vessels and adjacent regions at the indicated time points. Dots and bars indicate mean ± SD and n = 5 mice/group from two independent experiments. *P* value versus WT by two-tailed Mann-Whitney *U* test.

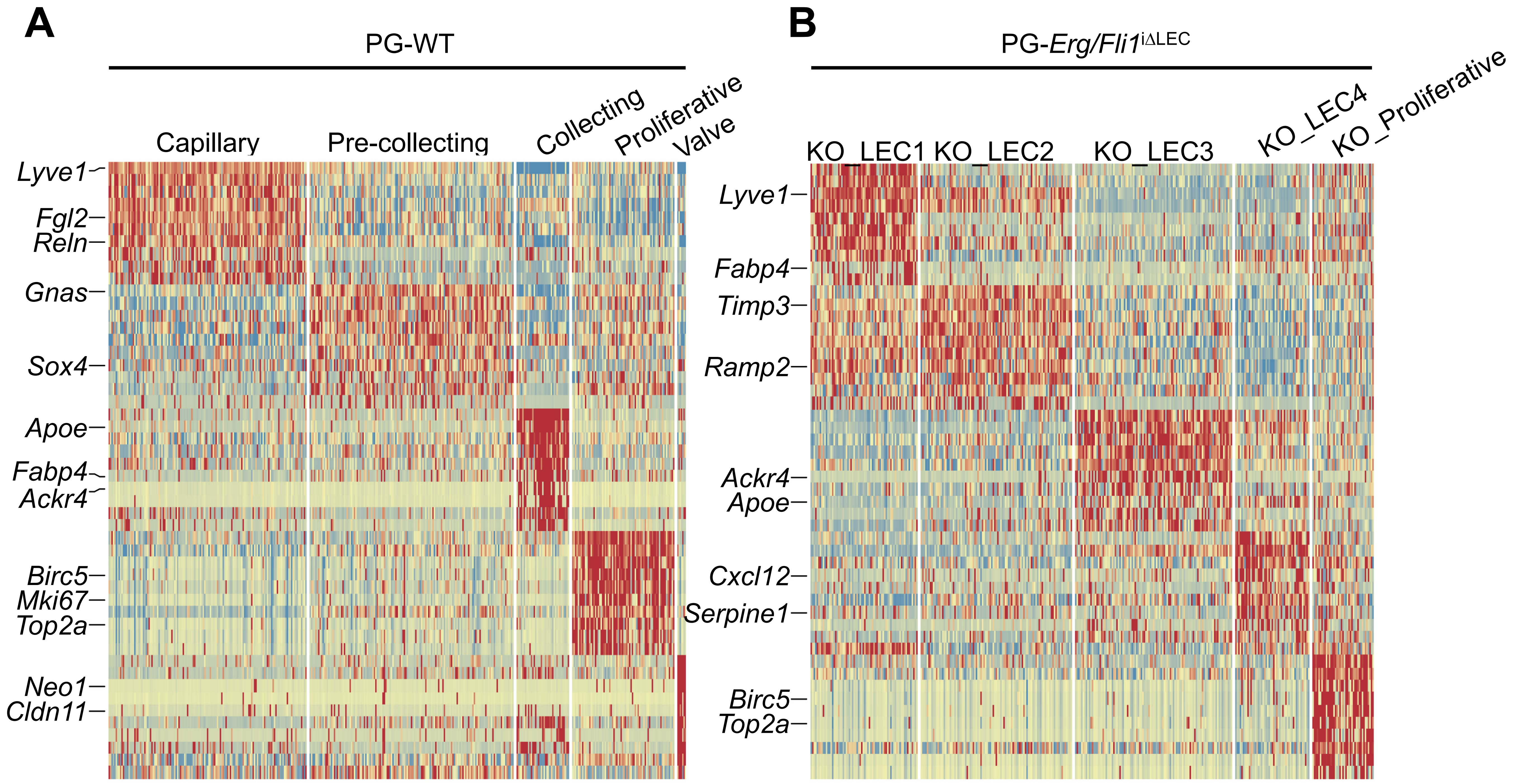

Supplemental Figure 5. Distinct transcriptomic profiles of mesenteric lymphatic subtypes in PG-WT or PG-*Erg/Fli1*$^{i\Delta LEC}$ mice

A,**B**, Heatmap visualizing scaled expression levels of top 10 differentially expressed genes in indicated clusters of mesenteric LEC in PG-WT (**A**) or PG-*Erg/Fli1*$^{i\Delta LEC}$ mice (**B**) at 2 weeks after the first Tmx injection.

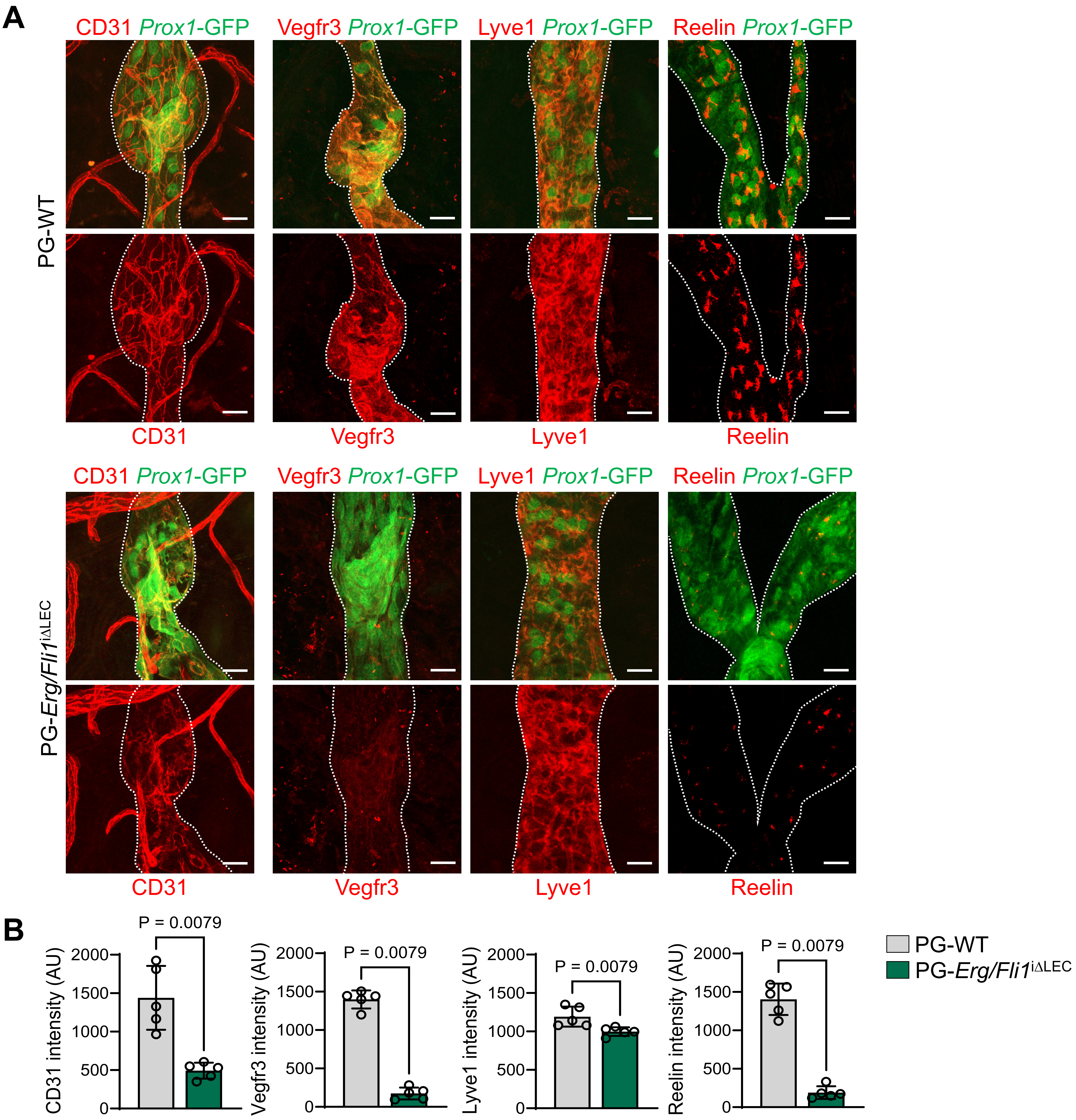

Supplemental Figure 6. Lymphatic signature proteins are reduced in the lymphatics of *Erg/Fli1*$^{i\Delta LEC}$ mice

A, B, Representative images and comparisons of protein levels of CD31, Vegfr3, Lyve1, and Reelin in the ear skin dermal lymphatics between PG-WT and PG-*Erg/Fli1*$^{i\Delta LEC}$ mice at 2 weeks after the first Tmx injection. Scale bars, 50 μm. Each dot indicates a value from one mouse and n = 5 mice/group from two independent experiments. Bars indicate mean ± SD and *P* value versus WT by two-tailed Mann-Whitney *U* test.

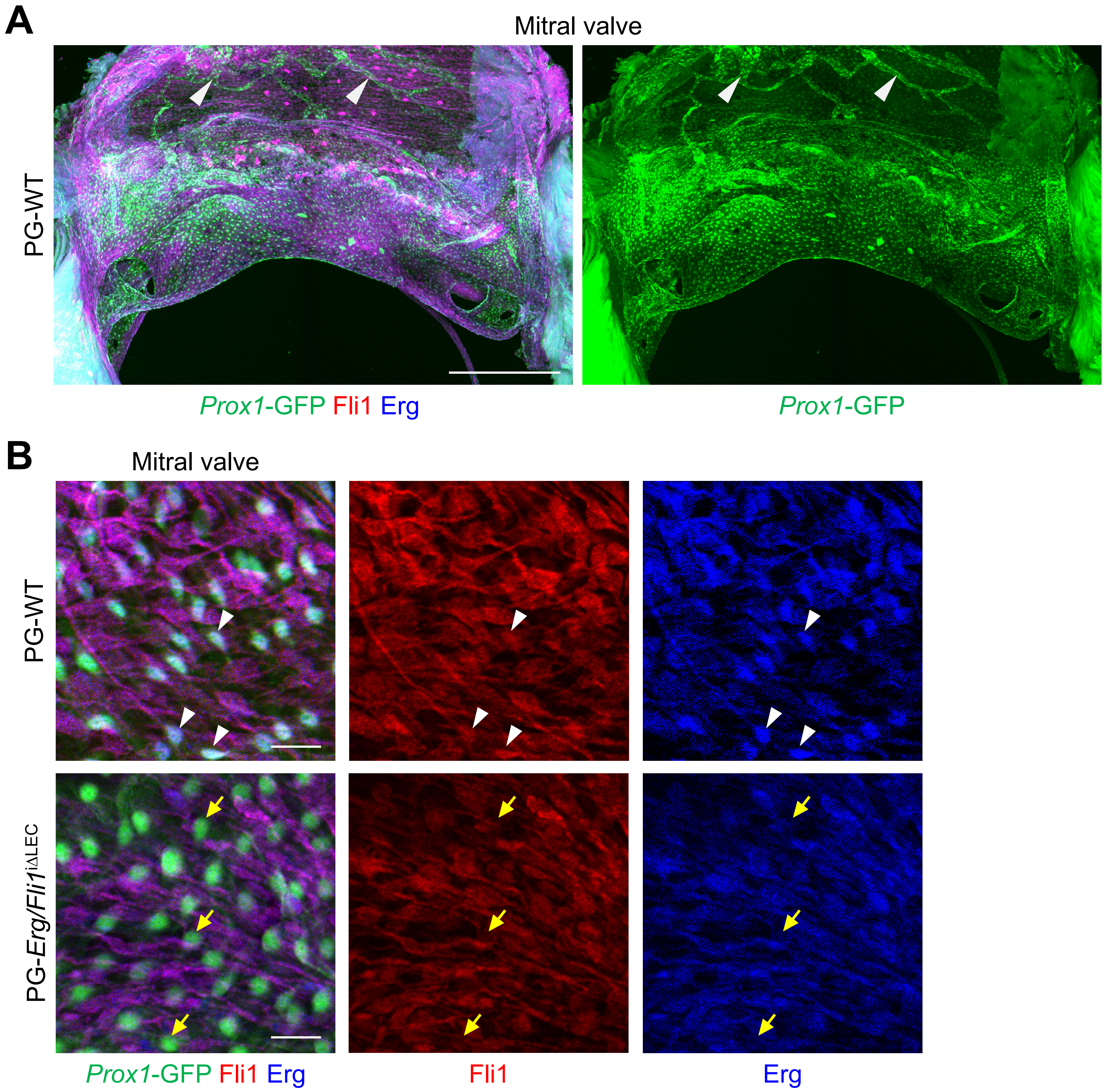

Supplemental Figure 7. Prox1^{+} cardiac valvular cells express Erg and Fli1

A, Immunofluorescence images of whole-mounted cardiac valve (mitral valve) in adult PG-WT mice. Cardiac valvular cells express Prox1. Note that Prox1+ lymphatics are present in the leaflet surface near the annulus area of the mitral valve (white arrowheads). Similar findings are shown from n = 5 mice/group from two independent experiments. Scale bars, 500 μm.

B, Immunofluorescence images of protein levels of Erg and Fli1 in whole-mounted cardiac valve (mitral valve) of PG-WT and PG-*Erg/Fli1*$^{i\Delta LEC}$ mice at 2 weeks after the first Tmx injection. Erg and Fli1 are expressed in Prox1^{+} cardiac valvular cells (white arrowheads). Erg and Fli1 were depleted in Prox1^{+} cardiac valvular cells in PG-*Erg/Fli1*$^{i\Delta LEC}$ mice (yellow arrows). Similar findings are shown from n = 5 mice/group from two independent experiments. Scale bars, 25 μm.

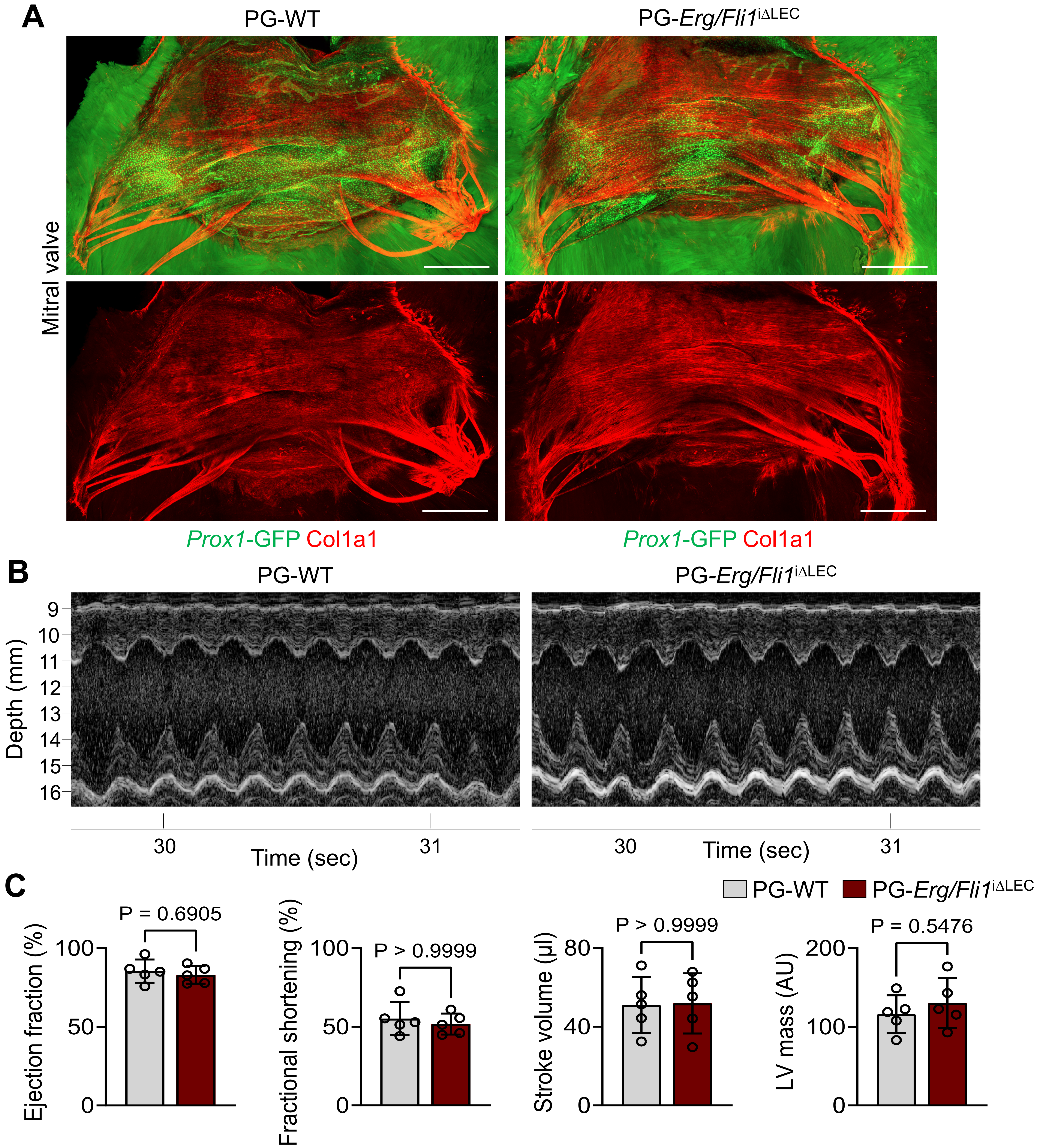

Supplemental Figure 8. No difference in cardiac valve structure and function in *Erg/Fli1*$^{i\Delta LEC}$ mice

A, Immunofluorescence images showing whole-mounted cardiac mitral valve in adult PG-WT and PG-*Erg/Fli1*$^{i\Delta LEC}$ mice at 2 weeks after the first of Tmx injection. Note no difference in mitral valve structure between the two groups. Similar findings are shown from n = 5 mice/group from two independent experiments. Scale bars, 500 µm.

B-C, Echocardiography M-mode images showing cardiac contraction across time in adult WT and *Erg/Fli1*$^{i\Delta LEC}$ mice at 10 days after the first Tmx injection. Ejection fraction, fractional shortening, stroke volume, and left ventricle mass were measured according to M-mode images. Each dot indicates a value from one mouse and n = 5 mice/group from two independent experiments. Bars indicate mean ± SD and *P* value *versus* PG-WT by two-tailed Mann-Whitney *U* test. AU = arbitrary unit.

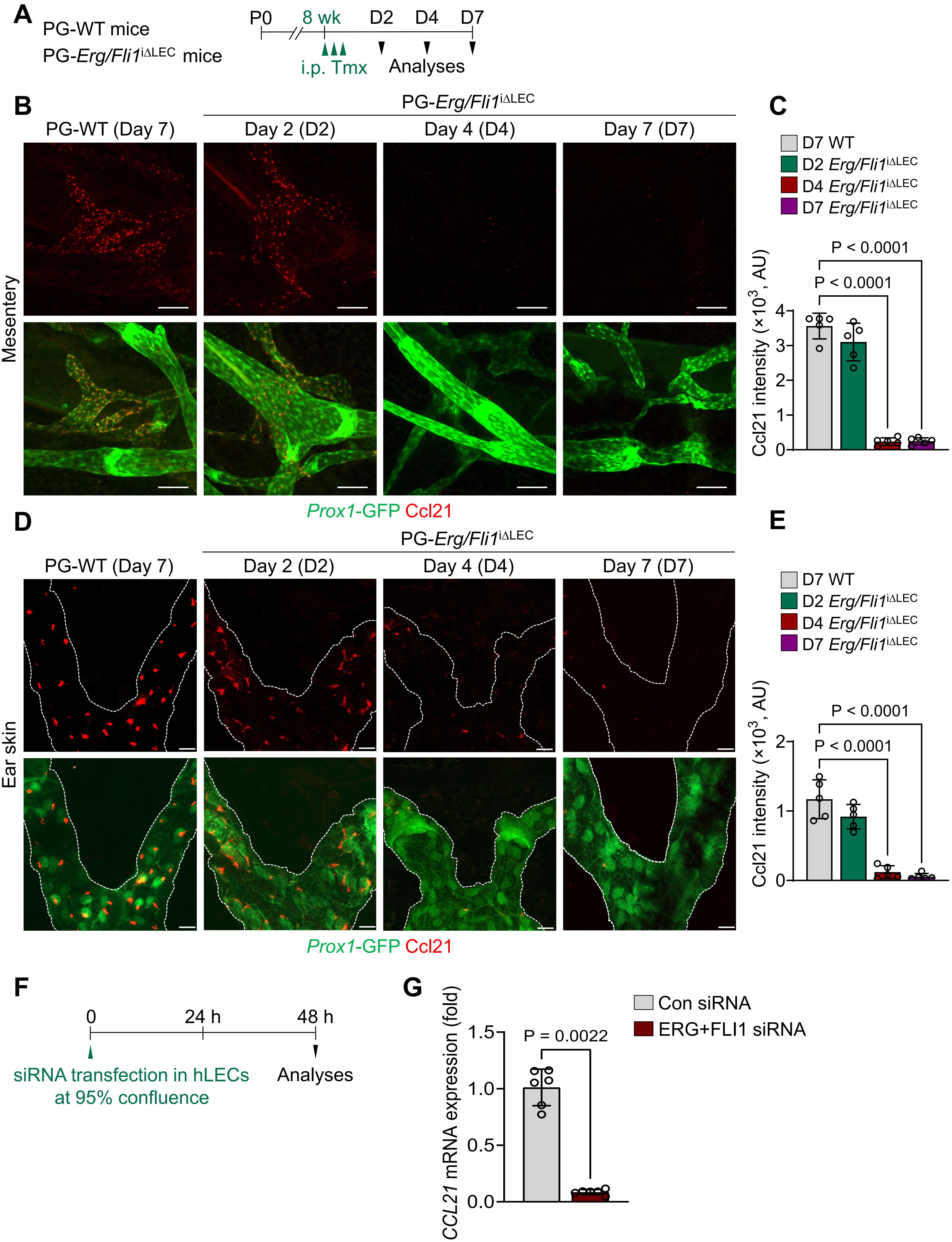

A
PG-WT mice
PG-Erg/Fli1iΔLEC mice
P0
8 wk
D2
D4
D7
i.p. Tmx
Analyses
B
PG-Erg/Fli1iΔLEC
PG-WT (Day 7)
Day 2 (D2)
Day 4 (D4)
Day 7 (D7)
Mesentery
Prox1-GFP Ccl21
C
D7 WT
D2 Erg/Fli1iΔLEC
D4 Erg/Fli1iΔLEC
D7 Erg/Fli1iΔLEC
P < 0.0001
P < 0.0001
Ccl21 intensity (×10³, AU)
D
PG-Erg/Fli1iΔLEC
PG-WT (Day 7)
Day 2 (D2)
Day 4 (D4)
Day 7 (D7)
Ear skin
Prox1-GFP Ccl21
E
D7 WT
D2 Erg/Fli1iΔLEC
D4 Erg/Fli1iΔLEC
D7 Erg/Fli1iΔLEC
P < 0.0001
P < 0.0001
Ccl21 intensity (×10³, AU)
F
0
24 h
48 h
siRNA transfection in hLECs
at 95% confluence
Analyses
G
Con siRNA
ERG+FLI1 siRNA
P = 0.0022
CCL21 mRNA expression (fold)

Supplemental Figure 9. Erg and Fli1 directly regulate *Ccl21a* expression in lymphatic endothelial cells

A-E, (**A**) Diagram depicting intraperitoneal Tmx injections to PG-WT and PG-*Erg/Fli1*$^{i\Delta LEC}$ mice for three consecutive days and timing of analyses. (**B**,**C**) Immunofluorescence images and comparisons of mesenteric lymphatics and their Ccl21 protein levels in PG-WT at day 7 and PG-*Erg/Fli1*$^{i\Delta LEC}$ mice at day 2, day 4, and day 7 after the first Tmx injection. Scale bars, 100 μm. Each dot indicates a value from one mouse and n = 5 mice/group from two independent experiments. Bars indicate mean ± SD and *P* value versus PG-WT by one-way ANOVA test followed by Tukey's *post-hoc* test.
(**D**,**E**) Immunofluorescence images and comparisons of ear skin lymphatics and their Ccl21 protein levels in PG-WT at day 7 and PG-*Erg/Fli1*$^{i\Delta LEC}$ mice at day 2, day 4, and day 7 after the first Tmx injection. Scale bars, 50 μm. White dotted lines outline the ear skin lymphatics. Each dot indicates a value from one mouse and n = 5 mice/group from two independent experiments. Bars indicate mean ± SD and *P* value versus PG-WT by one-way ANOVA test followed by Tukey's *post-hoc* test.

F,**G**, Diagram depicting siRNA transfection in primary cultured human LECs (hLECs) at 95% confluency and comparisons of *CCL21* mRNA expression at 48 h after transfection between control or ERG+FLI1 siRNA. Each dot indicates a value from one sample and n = 6 samples/group from two independent experiments. Bars indicate mean ± SD and *P* value versus Control siRNA by two-tailed Mann-Whitney *U* test. AU= arbitrary unit.

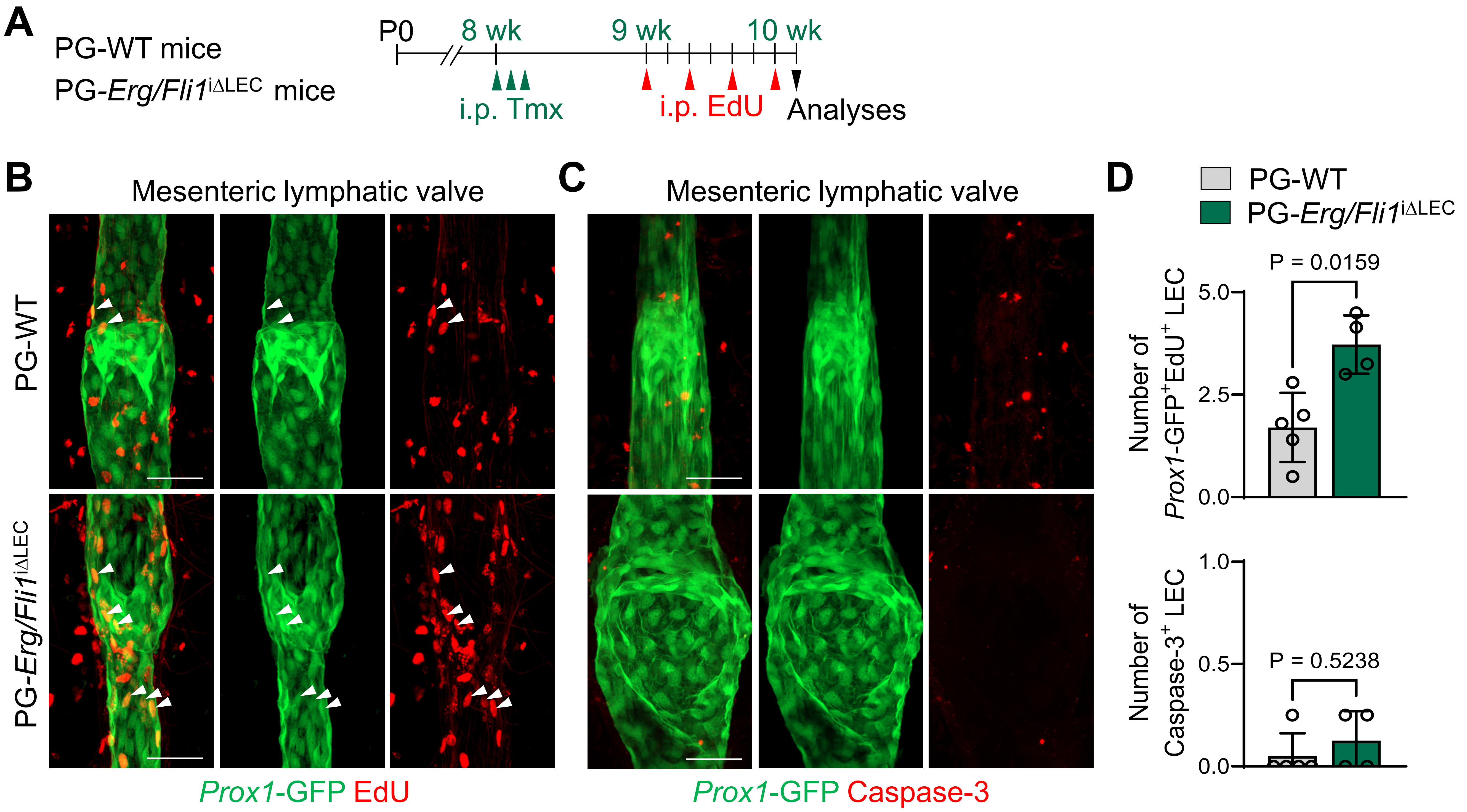

Supplemental Figure 10. Increased proliferation of mesenteric lymphatic valve LEC in *Erg/Fli1*$^{i\Delta LEC}$ mice

A, Diagram depicting showing intraperitoneal administrations of Tmx for three consecutive days, four intraperitoneal injections of EdU every alternative days one week later, and sampling for analyses in PG-WT and PG-*Erg/Fli1*$^{i\Delta LEC}$ mice

B-D, Representative images and comparisons of number of Prox1$^+$/EdU$^+$ LEC (white arrowheads) or Prox1$^+$ /caspase-3$^+$ LEC in mesenteric lymphatic valve between PG-WT and PG-*Erg/Fli1*$^{i\Delta LEC}$ mice. Similar findings are shown from n = 4-5 mice/group from two independent experiments. Scale bars, 50 μm. Bars indicate mean ± SD and *P* value versus WT by two-tailed Mann-Whitney *U* test.

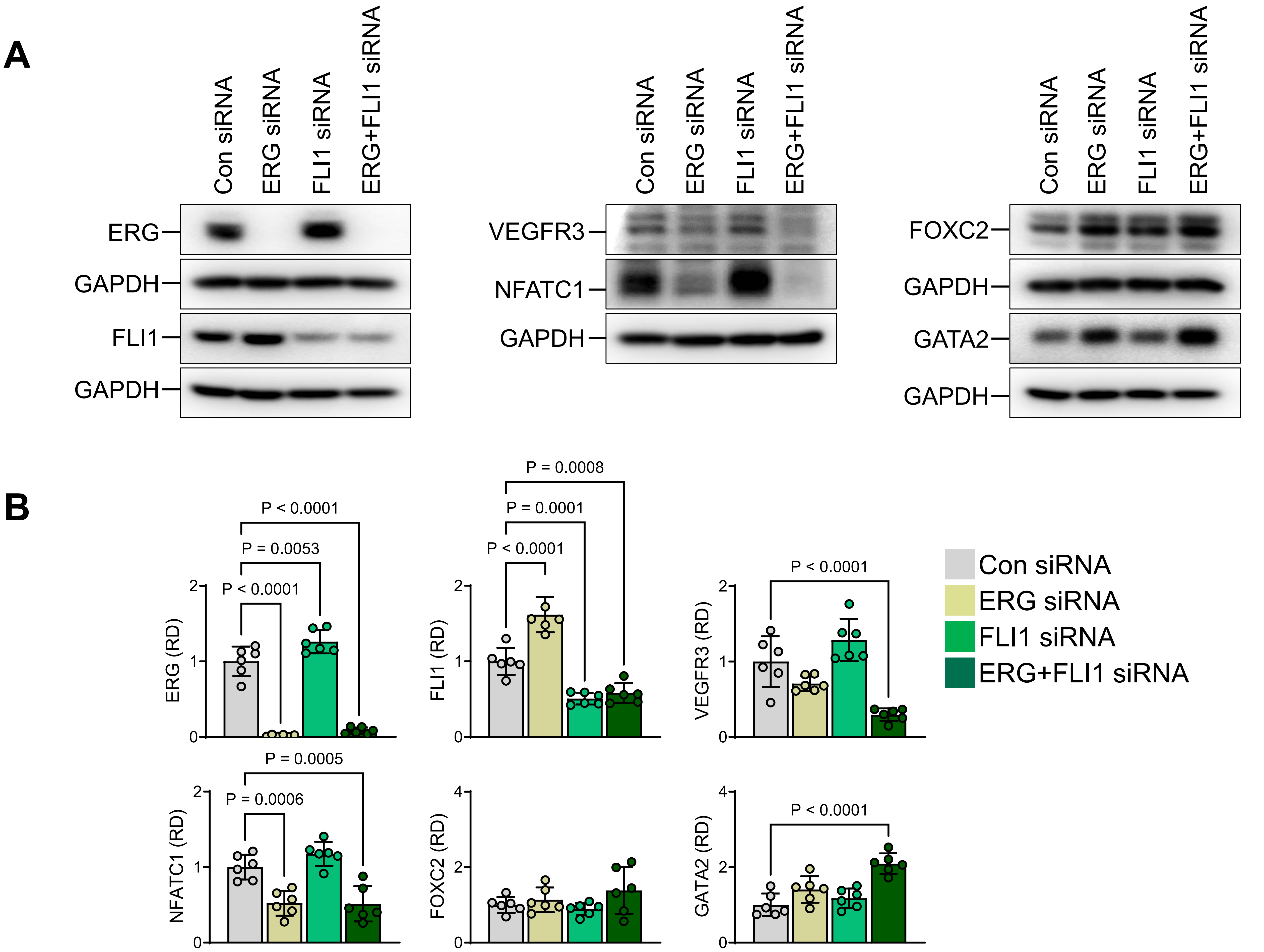

Supplemental Figure 11. ERG transcriptionally regulates NFATC1 expression in human LEC

A,**B**, Representative immunoblotting and comparisons of ERG, FLI1, VEGFR3, NFATC1, FOXC2, and GATA2 at 48 h after transfection of control, ERG, FLI1, or ERG+FLI1 siRNA in primary cultured human LEC. The same amount of protein loading in each lane is verified by immunoblotting of GAPDH. Each dot indicates a value from one sample and n = 6 samples/group from two independent experiments. Bars indicate mean ± SD and *P* value versus WT by one-way ANOVA test followed by Dunnett's *post-hoc* test.

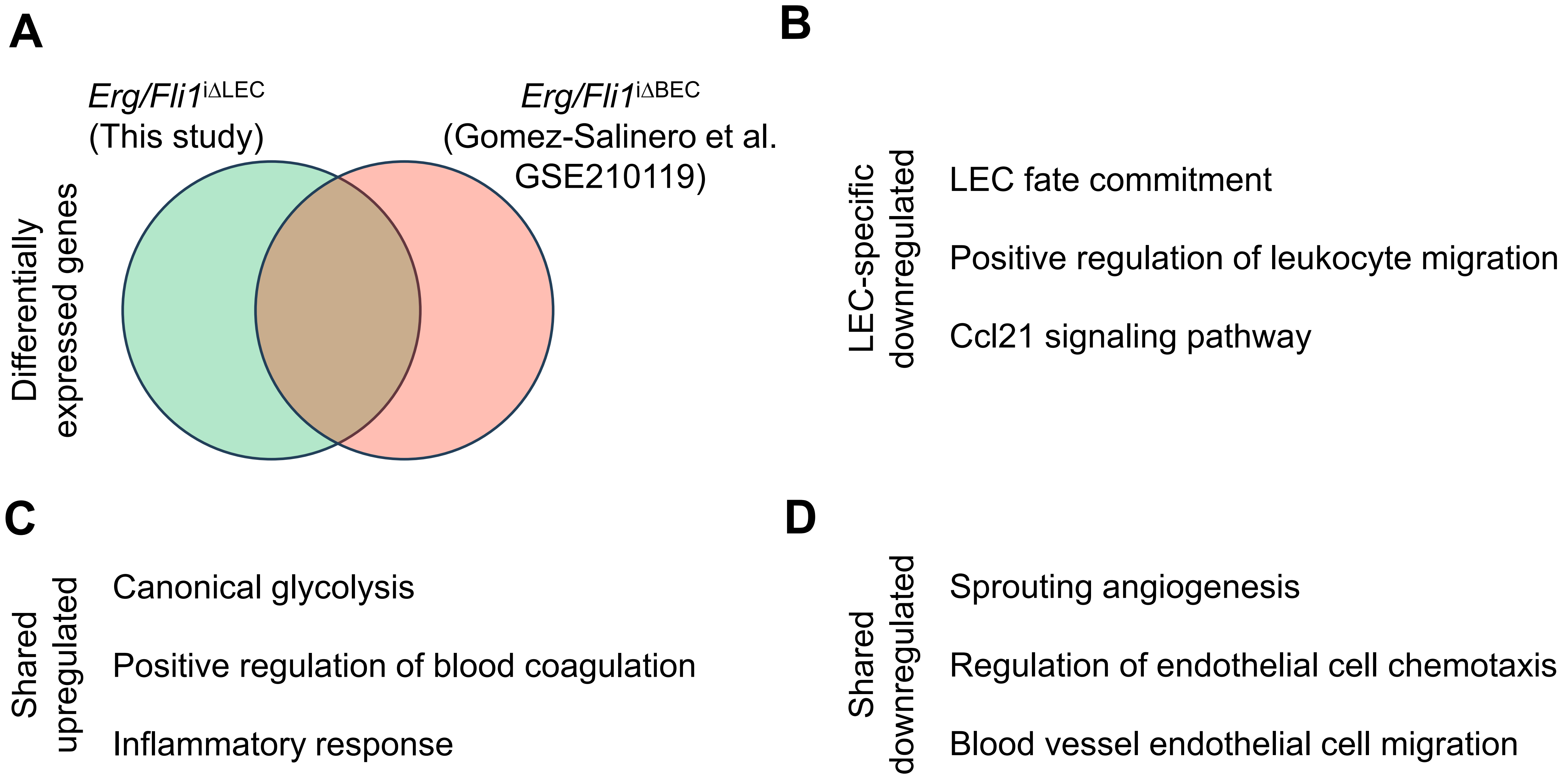

Supplemental Figure 12. Comparison of Erg/Fli1-regulated genes between blood and lymphatic endothelial cells

A, Venn diagram depicting comparison of differentially expressed genes after deletion of Erg and Fli1 in LEC or blood endothelial cells (BEC).

B-D, Bar plots showing significantly enriched gene ontology terms of LEC-specific downregulated genes, genes upregulated or downregulated in both LEC and BEC.

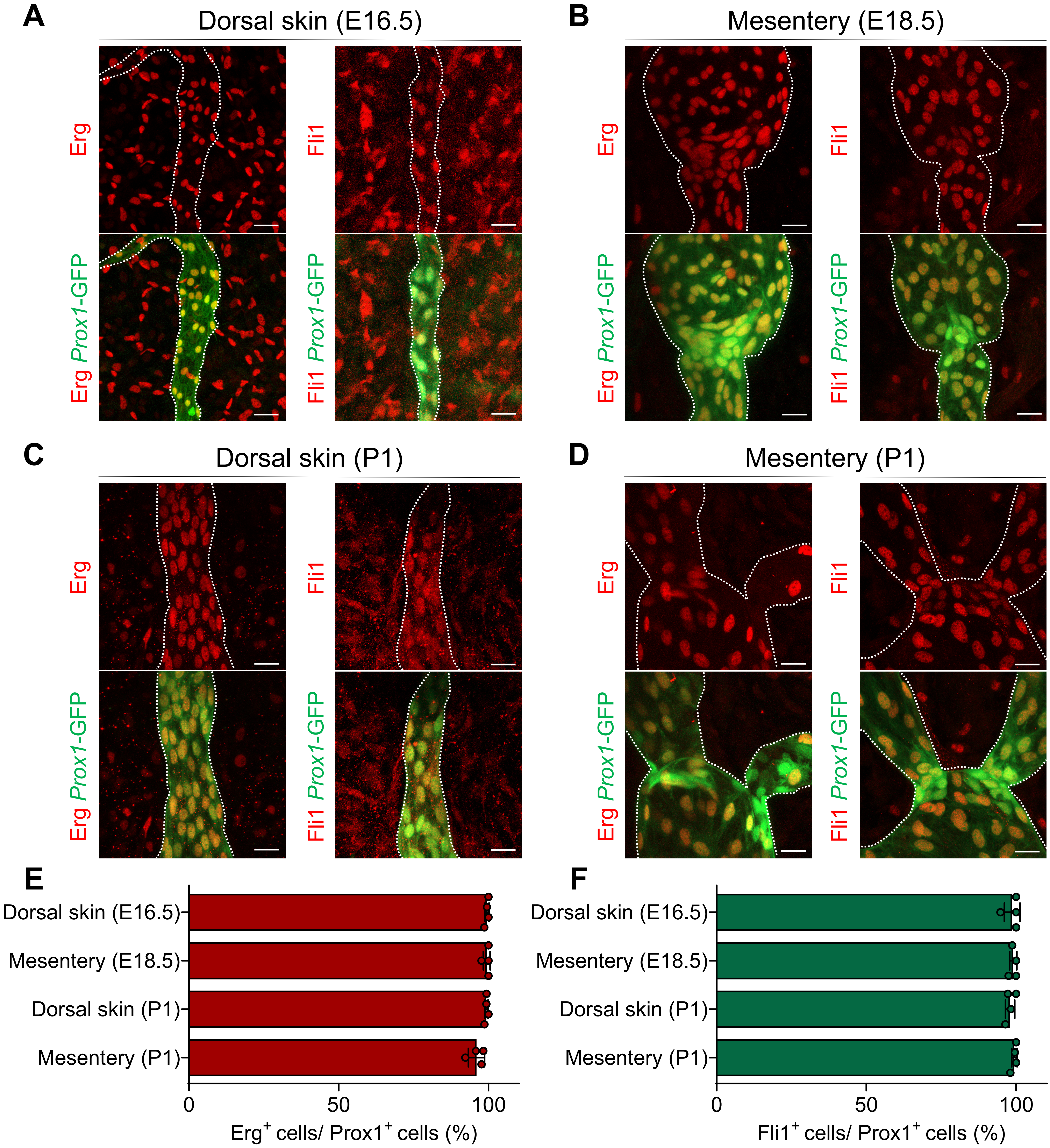

Supplemental Figure 13. Erg and Fli1 are present in LEC of dorsal dermal and mesenteric lymphatics during embryonic and postnatal periods

A-**F**, Representative images and percentages of Erg^{+} and Fli1^{+} LEC in the dorsal dermal or mesenteric lymphatics (white dotted lines) at embryonic day (E) 16.5, 18.5, or postnatal day (P)1. Scale bars, 20 µm. Each dot indicates a value from one mouse and n = 4 mice/group from two independent experiments. Vertical bars indicate mean ± SD.

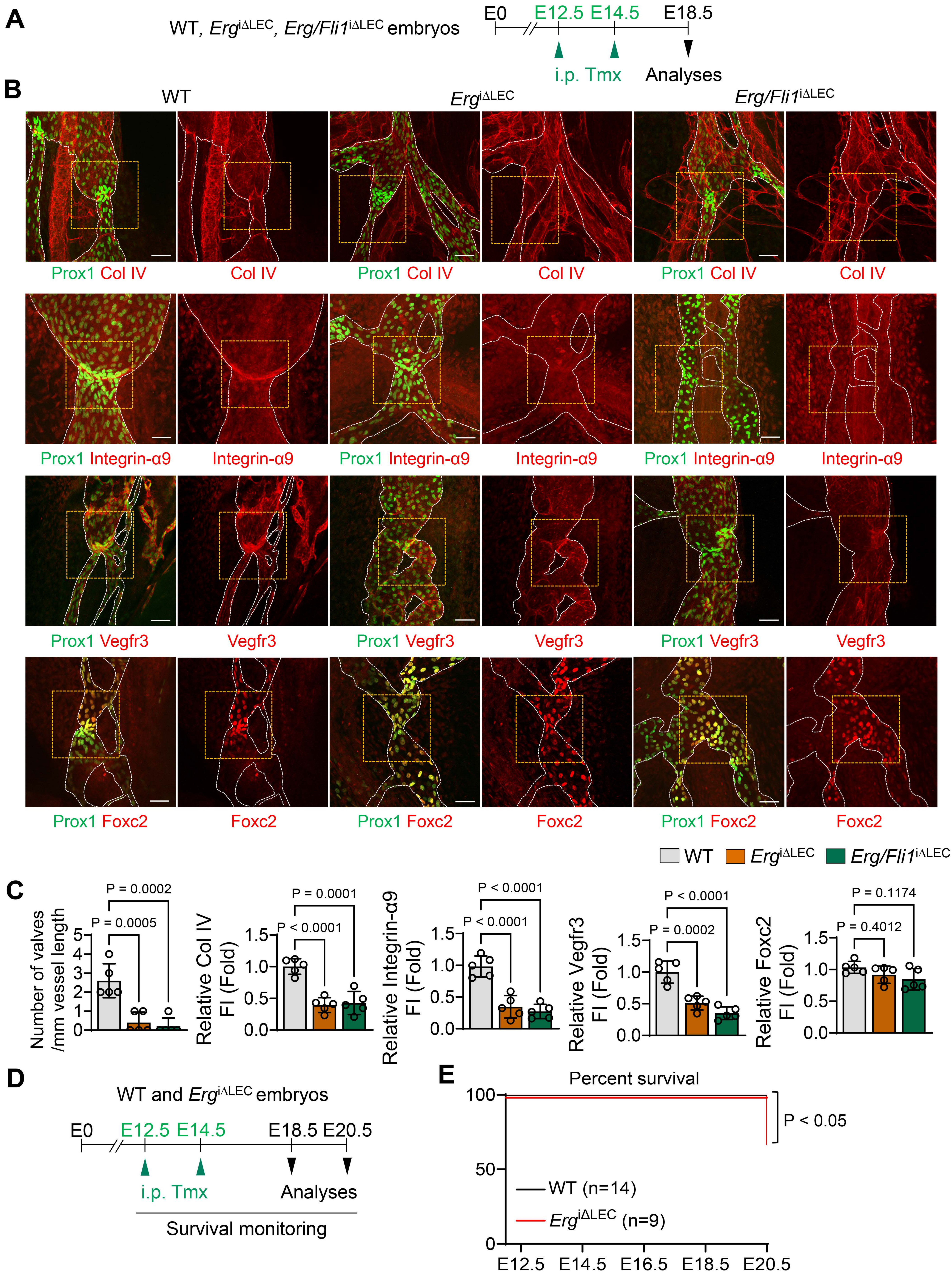

A
WT, Erg iΔLEC, Erg/Fli1 iΔLEC embryos
E0
E12.5
E14.5
E18.5
i.p. Tmx
Analyses
B
WT
Erg iΔLEC
Erg/Fli1 iΔLEC
Prox1 Col IV
Col IV
Prox1 Integrin-α9
Integrin-α9
Prox1 Vegfr3
Vegfr3
Prox1 Foxc2
Foxc2
WT
Erg iΔLEC
Erg/Fli1 iΔLEC
C
Number of valves /mm vessel length
P = 0.0002
P = 0.0005
Relative Col IV FI (Fold)
P = 0.0001
P < 0.0001
Relative Integrin-α9 FI (Fold)
P < 0.0001
P < 0.0001
Relative Vegfr3 FI (Fold)
P < 0.0001
P = 0.0002
Relative Foxc2 FI (Fold)
P = 0.1174
P = 0.4012
D
WT and Erg iΔLEC embryos
E0
E12.5
E14.5
E18.5
E20.5
i.p. Tmx
Analyses
Survival monitoring
E
Percent survival
P < 0.05
WT (n=14)
Erg iΔLEC (n=9)
E12.5
E14.5
E16.5
E18.5
E20.5

Supplemental Figure 14. Erg and Fli1 regulate lymphatic valve formation during embryonic development

A, Diagram depicting i.p. injection of Tmx to the pregnant mothers carrying WT, *Erg*$^{i\Delta LEC}$ and *Erg/Fli1*$^{i\Delta LEC}$ embryos at E12.5 and E14.5, and sampling at E18.5.

B,**C**, Representative images and comparisons of number of valves and expressions of valve markers, Col IV, Integrin-α9, Vegfr3, and Foxc2 in mesenteric lymphatics of WT, *Erg*$^{i\Delta LEC}$ and *Erg/Fli1*$^{i\Delta LEC}$ embryos. Yellow lined boxes indicate mesenteric lymphatic valve areas. Scale bars, 40 μm. Each dot indicates a value from one mouse and n = 5 mice/group from two independent experiments. Bars indicate mean ± SD and *P* value versus WT by one-way ANOVA test followed by Tukey's post-hoc test.

D, Diagram depicting i.p. injection of Tmx to the pregnant mothers carrying WT and *Erg*$^{i\Delta LEC}$ embryos at E12.5 and E14.5, and survival rate analysis at E18.5 and E20.5.

E, Kaplan-Meier curve showing survival rate of the indicated embryos following the initial Tmx administration. n = 9 or 14 mice/each group from two independent experiments. *P* value versus WT by Mantel-Cox comparison.

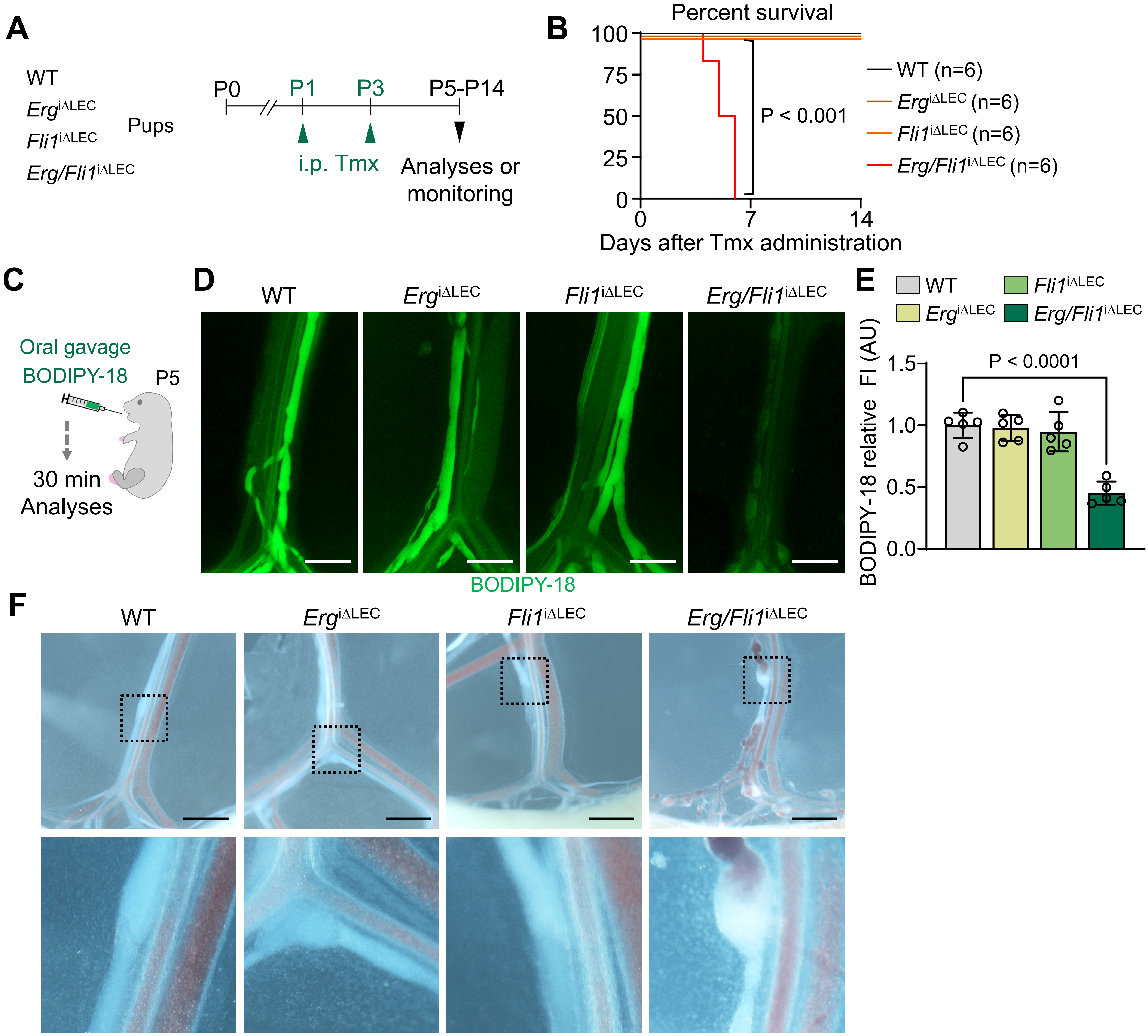

Supplemental Figure 15. Postnatal deletion of Erg and FLI1 impairs lymphatic drainage

A, Diagram depicting intraperitoneal injection of Tmx to WT, *Erg*$^{i\Delta LEC}$, *Fli1*$^{i\Delta LEC}$, and *Erg/Fli1*$^{i\Delta LEC}$ postnatal pups at postnatal day 1 (P1), and P3 and analyses at P5 or survival monitoring for 14 days .

B, Kaplan-Meier curve showing survival rate of the indicated pups following the initial Tmx administration. n = 6 mice/group from two independent experiments. *P* value versus WT by Mantel-Cox comparison.

C, Schematic diagram depicting oral gavage of BODIPY-18 to P5 mice and imaging of mesenteries 30 min later.

D,**E**, Representative images and comparison of absorbed BODIPY-18 in mesentery of indicated mice pups. Scale bars, 500 μm. Each dot indicates a value from one mouse and n =5 mice/group from two independent experiments. Bars indicate mean ± SD and *P* value versus WT by one-way ANOVA test followed by Dunnett's *post-hoc* test.

F, Representative images showing mesenteries of WT, *Erg*$^{i\Delta LEC}$, *Fli1*$^{i\Delta LEC}$, and *Erg/Fli1*$^{i\Delta LEC}$ at P5. Black dashed line boxes are enlarged in the below panels. Scale bars, 1 mm.

Supplemental Table 1. *CCL21* primer sets used for ChIP-qPCR

Primer		Primer sequence (5'-3')
***CCL21*_R1 (R1)**	Forward	TTGGGCTTTCCAGAAGGGCACTT
	Reverse	TTATGTTGTGGAGAAGCCACCCTCC
***CCL21*_R2 (R2)**	Forward	CCTGGTCTCAACAATGTGGCTGTGT
	Reverse	GAGATGGGTGTGTAGGTGAAGGATG
***CCL21*_R3 (R3)**	Forward	AAGACTCTGGGAACAACTGTACCC
	Reverse	GGGGAGAGGGCAGATCAGTTTT
***CCL21*_R4 (R4)**	Forward	CCTAAGGGACAGGCTTGCTTTG
	Reverse	CCCCGCAAAAAGATTCTGTCCC
***CCL21*_R5 (R5)**	Forward	CCACACTGTAATATTCAGAATGCCC
	Reverse	CTATGTCCAATATCTGGGCTTCCTC
***CCL21*_R6 (R6)**	Forward	CCACTTAGTTTGAGATAAGTGGGG
	Reverse	GTAGCTAGACACTTTCACTTCCC
***CCL21*_R7 (R7)**	Forward	CAGCATCTGGACAAGACACCATC
	Reverse	AGCCTTTGGAGCCCTTTCCTTTC
**Negative control (*RELA*(*P65*))**	Forward	TTCCATATCTGGCCACAAAA
	Reverse	TCCGTGTTCATGATTCATTG
