# Supplementary figures and images for: Cooperative ETS transcription factors are required for lymphatic endothelial cell integrity and resilience
Source: J Clin Invest. 2025 Dec 23;136(5):e196119. doi: 10.1172/JCI196119 (PMC13067931; doi:10.1172/JCI196119)

### Supplemental Figure 11A

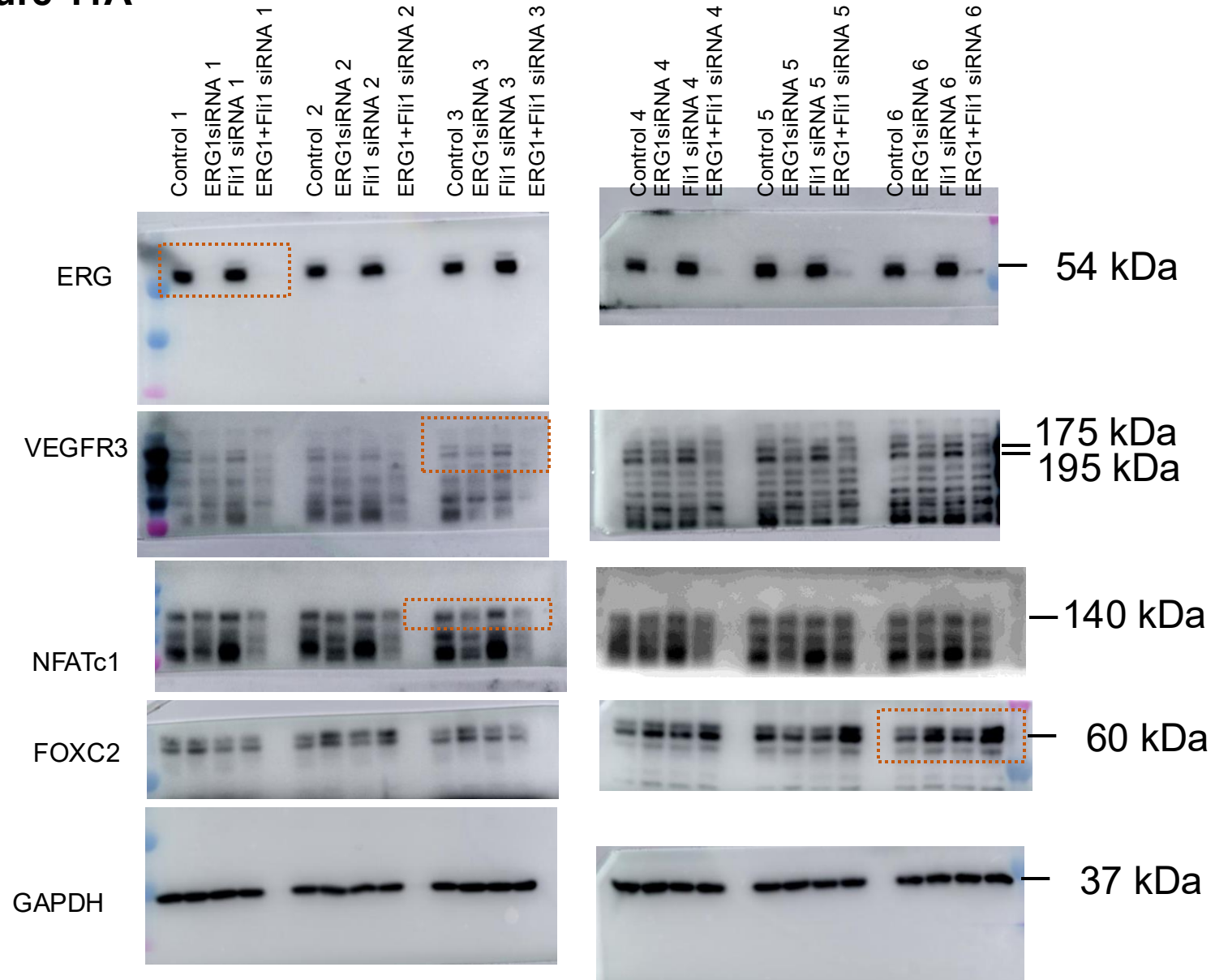

Supplemental Figure 11A

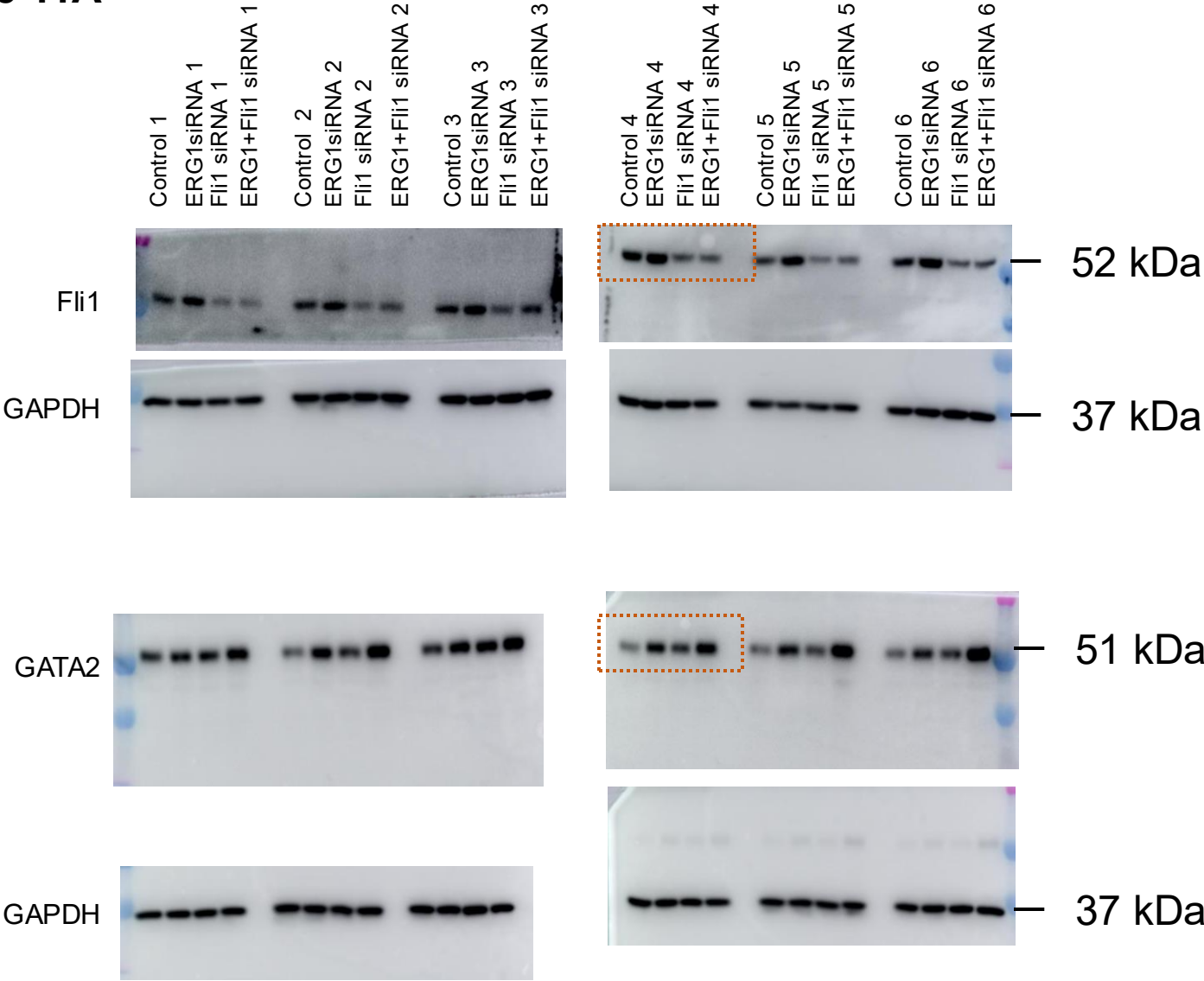

Supplement: Unedited blot and gel images [file jci-136-196119-s011.pdf]
